# To be or not to be a subspecies: description of *Saperda populnea lapponica* ssp. n. (Coleoptera, Cerambycidae) developing in downy willow (*Salix lapponum* L.)

**DOI:** 10.3897/zookeys.691.12880

**Published:** 2017-08-17

**Authors:** Henrik Wallin, Torstein Kvamme, Johannes Bergsten

**Affiliations:** 1 Department of Zoology, Swedish Museum of Natural History, P. O. Box 50007, SE-104 05 Stockholm, Sweden; 2 Norwegian Institute of Bioeconomy Research (NIBIO), P. O. Box 115, NO-1431 Ås, Norway

**Keywords:** Palaearctic region, Nearctic region, taxonomy, Cerambycidae, Lamiinae, *Saperda*, new subspecies, new synonyms, genitalia characters, *Salix lapponum*, subspecies definition, unified species concept

## Abstract

A new subspecies of the European cerambycid *Saperda populnea* (Linnaeus, 1758) is described: *Saperda populnea lapponica* **ssp. n.** based on specimens from Scandinavia. The male genitalia characters were examined and found to provide support for this separation, as well as differences in morphology, geographical distribution and bionomy. The preferred host tree for the nominate subspecies *S. populnea populnea* is *Populus tremula* L., whereas *S. populnea lapponica* **ssp. n.** is considered to be monophagous on *Salix lapponum* L. DNA sequence data of mitochondrial cytochrome oxidase subunit I (COI) was generated from Scandinavian specimens of *S. populnea populnea* and specimens representing *S. populnea lapponica* **ssp. n.** The two subspecies were not reciprocally monophyletic and genetic distances in COI were small. All synonyms of *S. populnea populnea* have been considered, and species similar to *S. populnea populnea* have been examined, and not found to be related to *S. populnea lapponica* **ssp. n.** A male lectotype has been designated for each of the two following synonyms: *Cerambyx decempunctatus* De Geer, 1775, and *Saperda salicis* Zetterstedt, 1818. The synonymised species from Asia, *S. balsamifera* (Motshulsky, 1860), is elevated to subspecies: *S. populnea balsamifera* **stat. n.** We end with a discussion on the definition of subspecies under the unified species concept.

## Introduction

The tribe Saperdini Mulsant, 1839 is extremely rich in species and consists of about 1000 species, mainly in the Oriental region (Bilý and Mehl 1989). The genus *Saperda* Fabricius, 1775, on the other hand, consists only of 42 species in the Holarctic region. In the Palaearctic region, 26 species and two subspecies are known (Aurivillius 1921, Löbl and Smetana 2010). In North America, 16 species and two subspecies were reported (Felt and Joutel 1904, Linsley and Chemsak 1995) but have recently been reduced to 15 species and one subspecies (Bezark 2016). There are no *Saperda* species from Europe also with Holarctic distribution, as currently defined (Bezark 2016). Only eight species are known from Europe (Bense 1995, Löbl and Smetana 2010), of which six species occur in Fennoscandia (Bilý and Mehl 1989, Silfverberg 2010).

Recently, there have been some taxonomic changes within the genus *Saperda*. *Saperda balsamifera* (Motschulsky, 1860) from east Palaearctic was listed as a separate species by Löbl and Smetana (2010). Shapovalov (2013) considered *S. balsamifera* to be synonymous with *S. populnea* (Linnaeus, 1758). Later, Danilevsky (2016) considered *S. balsamifera* to be a subspecies of *S. populnea*. The North American subspecies *S. populnea moesta* Le Conte, 1850 (Linsley and Chemsak 1995) was considered to be a valid species by Shapovalov (2013). The most recently described species of *Saperda* is *S. gilanense* Shapovalov, 2013 from Northern Iran.

**Figure 1.**
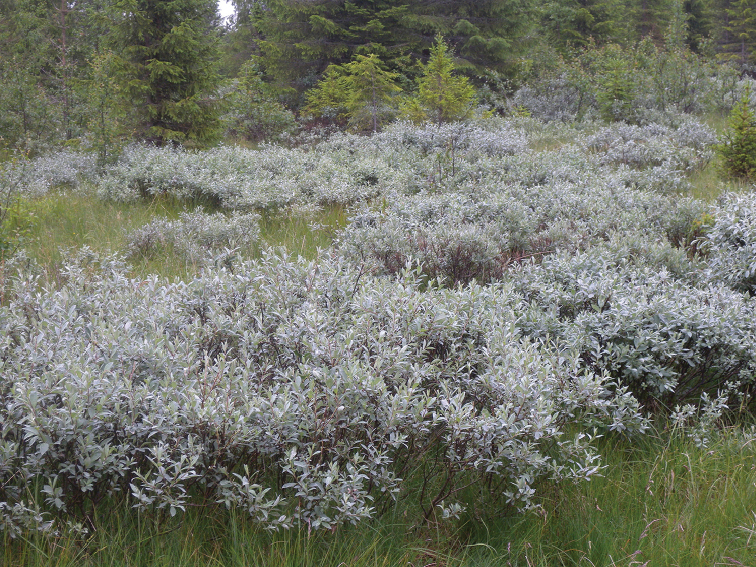
Habitat of *Saperda populnea lapponica* ssp. n., Trysil: Ljørdalen, Norway with an accumulation of downy willow (*Salix lapponum* L.) on a boreal and elevated boggy meadow.

Our study focus mainly on the northern populations of *S. populnea*, which have less dense and more greyish pubescence and found to be monophagous on downy willow, *Salix lapponum* L. Reared specimens were compared with the preserved type specimens of the southern populations which are larger and have denser and more orange-brown pubescence. The southern form was described by Linnaeus already in 1758. A large number of similar specimens from Scandinavia and other parts of Europe, often confirmed to have been collected on, or reared from, *Populus tremula* L. are included. *Saperda populnea lapponica* ssp. n., which we describe in this study from populations in the Fennoscandian mountains, has exclusively been reared from *Salix lapponum* (Fig. 1).

*Salix lapponum* is abundant at higher altitudes in the Scandinavian mountains, where the shrubs may reach a height of 1–2 m on moist areas such as bogs and swamps, but scarce or absent in the southern coastal areas (Hultén 1971, Elven 2005). Conversely, *Populus tremula* is scarce or absent in mountain areas in Scandinavia where *S. lapponum* is most abundant (Hultén and Fries 1986). *S. lapponum* is distributed in northern Europe and eastwards into Siberia, approximately to the Jenisej Valley as well as in northern Scotland (Hultén and Fries 1986). We have no information on *S. populnea lapponica* ssp. n. or *S. populnea populnea* attacking *Salix lapponum* in Scotland, or elsewhere in the UK.

We have not been able to find any attacks on, or specimens reared from, any other *Salix* species in areas where *Saperda populnea lapponica* ssp. n. is common. All the specimens from Scandinavia have been recorded at localities where *Salix lapponum* is abundant (Fig. 1). We therefore consider *S. populnea lapponica* ssp. n. to be monophagous on *Salix lapponum* in Scandinavia. Taxonomic position of *Salix lapponum* is rather isolated from other *Salix* species in the Palaearctic Region. It is placed in the subgenus Vetrix, in the section Villosae. This section only includes the nearest relative *Salix alaxensis* (Andersson) Coville from North America, apart from *Salix lapponum* (Reidar Elven pers.com.). *Salix lapponum* is known to hybridize with many other species. Both hybrids and triple hybrids as well as diploids/polyploids are known (Jonsell 2000), but we do not know if the hybrids or polyploids are used as host trees. *Salix lapponum* is also well known to be a “mild tasting” food for herbivores, due to a low content of phenolic components (Elven 2005). *Populus tremula* is absent in the spots where we found *Salix lapponum*. However, several *Salix* species occur in these biotopes. *Populus tremula* requires drier soil, and is therefore not found in the same biotopes as *S. lapponum* (Reidar Elven pers. com.).

We have also made a comparison with other *Saperda* species from Europe, Asia (Siberia) and North America, with special emphasis on related species in the subgenus Compsidia Mulsant, 1839. The presented taxonomic study is based on examination of morphological characters as well as studies of the genitalia. We also use two different fragments of the mitochondrial gene cytochrome oxidase subunit I (COI) to test for reciprocal monophyly and calculate genetic distances. We adhere to the unified species concept (de Quieroz 2007) and define species as separately evolving metapopulation lineages. There are several lines of evidence that can be used to show that two lineages are separately evolving, such as intrinsic reproductive isolation, ecological niche differentiation, phenotypically divergent and diagnosable and reciprocally monophyletic in genetic markers. However, none of these are part of the species definition (de Quieroz 2007). We define subspecies under the unified species concept as potentially incipient species in allopatry or parapatry that are diagnosable by at least one presumably heritable trait (see further under discussion).

## Methods

### Taxon sampling for DNA

All 17 available sequences for *Saperda populnea* in Bold and Genbank were downloaded. Apart from one sequence of mitochondrial ribosomal 16s, the remaining 16 were of mitochondrial cytochrome oxidase subunit I (COI). Of these one turned out to be misidentified (KF247304), one was of the 3-prime (“pat-jerry”) fragment of COI and 14 were of the 5-prime (LCO-HCO) barcode fragment of COI. Thirteen of these were from Finland and the FINBOL Barcoding project and had been released by Pentinsaari et al. (2014). The last (KM286402) was from a specimen from the French Alps. We combined these 14 sequences with available sequences of the same fragment of COI from other *Saperda* species to analyse the gene tree topology and intraspecific variation. The downloaded sequences were aligned with Clustal X (Larkin et al. 2007) under default settings. The 13 *Saperda populnea* specimens from Finland included two specimens from Lappish Finland and were of the smaller less hairy form that fitted our concept of what we will hereafter refer to as *S. populnea lapponica* ssp. n. (see taxonomy part), based on photos. However, the sequences for all the Finnish material were shorter (407 bp) than normal barcode fragments (full 658 bp; minimum barcode standards >500 bp). After examining the alignment, it turned out that they only differed at two positions (including the French specimen). We therefore decided to aim for the longer 3-prime end 825 bp fragment of CO1 for the new material.

New material of both *S. populnea lapponica* ssp. n. and *S. populnea populnea* was collected as larva from the host plants *Populus tremula* and *Salix lapponum* in Sweden and Norway 2009-2013 (Tab. 1). We also collected new material of related species of the genus *Saperda*, both as larvae and adults. In addition, we extracted a number of dry-pinned adult specimens from the collections at the Swedish Museum of Natural History (NHRS), Stockholm, Sweden. These ranged in collection dates from 1948–1994 and included material from the two important donations of Lars Huggert and Stig Lundberg (Tab. 1). All DNA vouchers are kept at NHRS.

### Molecular laboratory protocols

DNA from imagines was extracted from adults using 1 leg, 2 legs, thoracic muscle tissue, or head and prothorax. When DNA from larvae was extracted, tissue from tergites or sternites was used. Extraction of DNA was done by using either the Quiagen tissue kit, or a GeneMole robot (Tab. 1), following standard protocols for both apart from using 20ul of DTT (Dithiothreitol). DTT may improve DNA extraction of material with degraded DNA as with the dry-pinned 20-70 years old samples. For fresh alcohol samples we amplified the COI fragment using primers “PatDyt” (TCATTGCACTAATCTGCCATATTAG; Isambert et al. 2011) and “Jerry” (CAACATTTATTTTGATTTTTTGG; Simon et al. 1994). When older material was used we attempted to amplify DNA in two or three overlapping fragments, each about 400-450 bp long using primer pairs Jerry - Hal450rw (GGAAATCATTGAATAAATCCAGCT), Hal200fw (CTGCAACAATAATCATTGCTGTTC) - Hal600rw (AAGCATCTGGATAATCAGAATATC) and Hal450fw (AGCTGGATTTATTCAATGATTTCC) - PatDyt. The first and third fragment overlaps at the 450fw/450rw primer-binding site which the second fragment covers. These primer pairs were originally designed by JB to amplify this COI part in two or three fragments from degraded DNA of Haliplidae. But it turns out that it also works for other families of Coleoptera.

Ready-ToGo™ PCR beads (Amersham Biosciences) were used in all PCR recations and 2-4ul of DNA. The longer fragments were amplified under the following conditions: 95C for 5min followed by 40 cycles of 95C for 30s, 50C for 30s and 72C for 60s and a final extension period of 72C for 8min. The shorter fragments were amplified under the same conditions or with a shorter extension time (72C 50s). In second trials with samples that failed the first time, the annealing temperature was lowered to 47C. PCR reactions were purified with Exonuclease I and FastAP (Fermentas) and sequenced with a BigDye™ Terminator ver. 1.1 Cycle Sequencing Kit (Applied Biosystems), cleaned with a DyeEx 96 kit (QIAGEN) and ran on an ABI Prism 3100 Genetic Analyzer (Applied Biosystems).

### Molecular analyses

Sequence chromatograms were edited in SEQUENCHER (Gene Codes Corporation). Contigs were created of the forward and reverse reads and of the two or three overlapping fragments for the older material. Sequences were exported in fasta format after primers had been removed and aligned using CLUSTALX 2.0 (Larkin et al. 2007). There were no gaps in the alignment.

We calculated genetic distances under the Kimura 2-parameter model using MESQUITE (Maddison and Maddison 2017). For both 5-prime and 3-prime datasets we performed a Bayesian clock analysis in BEAST 1.8.4 (Drummond et al. 2012). Ultrametric genetrees were inferred under a HKY+I+G substitution model with a strict clock model for branch lengths and allowed each codon position its own relative substitution rate. A constant size coalescent tree prior was used, as it was the tree topology and branch length within *Saperda populnea* that was of interest, not the relationship to other *Saperda* species. The MCMC analysis was run for one million generations, sampled every 1000 generations. A maximum clade credibility tree with median node heights and clade support values was computed using TREE ANNOTATOR (part of the Beast package). TRACER 1.6 (Rambaut et al. 2014) was used to control the performance of the runs.

### Morphological study

Our study includes descriptions of the sclerotised parts of the male terminalia: the aedeagus, endophallus with the sclerites inside the median phallomere and the internal sac, tegmen with parameres and median lobe, and tergite VIII. The internal sac of the males was embedded in glycerol and photographed using a regular light microscope. This method is described in detail by Wallin et al. (2009, 2012, 2013). The studies of the female terminalia included tignum, tergite VIII and the spermathecal capsule. Other parts of the male genitalia and also the female genitalia were dry mounted. The terminology used is based on Lingafelter and Hoebeke (2002), Hubweber and Schmitt (2010), Yamasako and Ohbayashi (2011), Lin et al. (2009), Slipiñski and Escalona (2013), Wallin et al. (2014) and Wallin and Kvamme (2015).

We maintain the use of the internal sac (part of the median phallomere), since it has been frequently used in the past (*cf.* Wallin et al. 2013). The sclerites inside the internal sac may vary considerably between species and have been found to be very useful when describing species of *Leiopus* (Wallin et al. 2012), *Monochamus* (Wallin et al. 2013), *Sybra* species (Weigel and Skale 2009) and species of *Nemophas* (Wallin et al. 2014). However, such sclerites are less variable in the genera *Saperda* and *Stenostola* since they mostly consist of three long shafts (without extensions), varying little in size and shape (Sama 2008). Hind wing morphology follows Lingafelter and Hoebeke (2002).

Male genitalia photos were taken using an Olympus SZX 10 UC 30 camera attached to a Zeiss microscope and operated via the software ANALYSIS docum and Olympus Soft Imaging Solutions GmbH Version 5.1 (Build 2677). No stacking was used on these images. Habitus photos were taken using a Canon EOS 5D Mark II DSLR camera with a Canon MP-E 65mm f/2.8 1–5× macro lens and a Canon MT-24EX Macro Twin Lite flash with custom-made light diffusors. The camera was mounted on a motorized Stackshot rail (Cognisys) and operated via the software ZERENE STACKER (Zerene Systems) that was also used for stacking the images. Measurement data of body length (BL) and the ratio (BL/BW) between body length and maximum body width (BW) was first tested for normality with a Shapiro-Wilk normality test in R (R Core Team, 2016). Normality was rejected for at least one species x sex category for both measurements. We therefore used the non-parametric Wilcoxon rank sum test of independent samples (also known as the Mann-Whitney U test, or the Wilcoxon-Mann-Whitney test). In order to evaluate the variation between species, we have also included specimens from North America and Asia.

**Table 1.** Metadata for specimens included in the molecular analysis. Column four gives GenBank accession numbers.

Species	Extr. ID	Ext method	CO1 Acc	Stage	from	Country, province, locality	Date	Leg.
*Saperda p. lapponica*	JB941	Qiagen	MF491465	larva	*Salix lapponum*	Norway, Hedmark, Ljørdalen	27.06.2013	Torstein Kvamme
*Saperda p. lapponica*	JB942	Qiagen	MF491467	larva	*Salix lapponum*	Norway, Hedmark, Engerdal	27.06.2013	Torstein Kvamme
*Saperda p. lapponica*	JB946	Qiagen	MF491463	larva	*Salix lapponum*	Norway, Hedmark, Ljørdalen	27.06.2013	Torstein Kvamme
*Saperda p. lapponica*	JB949	Qiagen	MF491468	larva	*Salix lapponum*	Sweden, Lule lappmark, Kiruna	24.06.2013	Torstein Kvamme
*Saperda p. lapponica*	JB950	Qiagen	MF491462	larva	*Salix lapponum*	Norway, Hedmark, Trysil	27.06.2013	Torstein Kvamme
*Saperda p. lapponica*	JB016	GeneMole	Failed	adult		Sweden, Torne Lappmark, Silkimuotka	28.VI.1948	N. Höglund
*Saperda p. lapponica*	JB017	GeneMole	Failed	adult		Sweden, Torne Lappmark, Silkimuotka	28.VI.1948	N. Höglund
*Saperda p. lapponica*	JB021(JB250)	GeneMole	MF491469	adult		Sweden, Åsele Lappmark, Kittelfjäll	28.VI.1972	T-E Leiler
*Saperda p. lapponica*	JB022(JB249)	GeneMole	MF491461	adult		Sweden, Torne Lappmark, Soppero	30.VI.1980	Stig Lundberg
*Saperda p. lapponica*	JB023(JB248)	GeneMole	Failed	adult		Sweden, Torne Lappmark, Soppero	15.VI.1968	Stig Lundberg
*Saperda p. lapponica*	JB024(JB251)	GeneMole	MF491460	adult		Sweden, Lule Lappmark, Messaure	14.VII.1971	S. Lundberg & T. Müller
*Saperda p. populnea*	JB945	Qiagen	MF491471	larva	*Populus tremula*	Sweden, Uppland, Uppsala	07.2013	Henrik Wallin
*Saperda p. populnea*	JB018(JB247)	GeneMole	MF491471	adult		Sweden, Öland, Räpplinge	03.V.1976	Bert Gustafsson
*Saperda p. populnea*	JB019(JB246)	GeneMole	MF491466	adult	*Salix* sp.	Sweden, Småland, Åseda	26.XII.1974	Bert Gustafsson
*Saperda p. populnea*	JB020(JB245)	GeneMole	MF491470	adult	*Salix* sp.	Sweden, Uppland, Uppsala	01.V.1984	Stig Lundberg
*Saperda p. populnea*	JB025(JB252)	GeneMole	MF491459	adult		Sweden, Norrbotten, Kalix	30.VI.1994	S. Lundberg & T. Müller
*Saperda p. populnea*	JB026	GeneMole	Failed	adult		Sweden, Västerbotten, Umeå	09.V.1969	Lars Huggert
*Saperda p. populnea*	JB027	GeneMole	Failed	adult		Sweden, Halland, Släp	02.V.1965	Lars Huggert
*Saperda p. populnea*	JB028	GeneMole	Failed	adult		Sweden, Västergötland, Amundön	31.12.1968	Lars Huggert
*Saperda p. populnea*	JB029	GeneMole	MF491472	larva	*Populus tremula*	Sweden, Uppland, Uppsala	05.2009	Henrik Wallin
*Saperda scalaris*	JB030	GeneMole	MF491473	adult	*Quercus robur*	Sweden, Uppland, Knutby	05.2009	Henrik Wallin
*Saperda similis*	JB938(RB122)	Qiagen	MF491458	larva	*Salix caprea*	Sweden, Uppland, Uppsala	07.2013	Henrik Wallin
*Saperda carcharias*	JB944	Qiagen	MF491456	larva	*Populus tremula*	Sweden, Uppland, Knutby	07.2013	Henrik Wallin
*Saperda carcharias*	JB031	GeneMole	MF491457	adult		Sweden, Södermanland, Haninge	20.IX.2009	Julio Ferrer
*Saperda moesta*	JB939	Qiagen	Failed	adult	*Populus balsamifera*	Canada, Ontario, Ottawa	07.07.1961	S.D. Hicks
*Saperda tulari*	JB943	Qiagen	Failed	adult	*Populus fremontii*	USA, California, Turlock	24.05.1955	R.R. Snelling
*Oberea oculata*	JB948	Qiagen	MF491455	larva	*Salix caprea*	Sweden, Uppland, Knutby	07.2013	Henrik Wallin

### Rearing of adult beetles

Stems and branches were cut from shrubs of *Salix lapponum* at localities where the host plant was abundant. Only host material with visible attacks was collected. At one locality near the road, the shrubs had been cut by ditch cleaning machines and infested branches were collected from the ground. The infested stems and branches of *Salix lapponum* were placed in rearing cabinets stored at room temperature. Most of the material was collected from mid-May to the beginning of June, shortly after snowmelt.

### Nomenclature applied

The species nomenclature follows Linsley and Chemsak (1995) and Löbl and Smetana (2010).

Specific information on examined specimens is mentioned under each species in the section “Taxonomy”. The dates and other information were copied from the labels. In some cases, additional information provided by collectors has been added.

### Abbreviations

**AMNH** American Museum of Natural History, New York, USA

**BPBM** Bernice Pauahi Bishop Museum, Honolulu, USA

**CAEL** Collection Arne E. Laugsand

**CBE** Collection Bengt Ehnström, Nås, Sweden

**CCH** Collection Carolus Holzschuh, Villach, Austria

**CHW** Collection Henrik Wallin, Uppsala, Sweden

**CMD** Collection Michail Danilevsky, Moscow, Russia

**COS** Collection Ove Sørlibråten, Mysen, Norway

**CPKS** Collection Per Kristian Solevåg, Lier, Norway

**CPS** Collection Pesarini & Sabbadini, Milano, Italy

**CRP** Collection Roger Petterson, Laxbacken, Sweden

**CTK** Collection Torstein Kvamme, Ås, Norway

**CUN** Collection Ulf Nylander, Gävle, Sweden

**CÅL** Collection Åke Lindelöw, Uppsala, Sweden

**GNM** Göteborg Natural History Museum, Gothenburg, Sweden

**LINN** Collection of The Linnean Society of London, London, UK

**MZH** Helsinki Natural History Museum, Helsinki, Finland

**MCZ** Museum of Comparative Zoology Harvard University, Cambridge, Massachusetts, USA

**MNHN** Muséum National d’Histoire Naturelle, Paris, France

**ZMUB** Natural History Collections, Bergen Museum, University of Bergen, Norway

**NHMO** Natural History Museum Oslo, University of Oslo, Norway

**NHRS** Swedish Museum of Natural History, Stockholm, Sweden

**NIBIO** Norwegian Institute of Bioeconomy Research, Ås, Norway

**ZMUO** University of Oulu, Finland

**UUZM** Museum of Evolution–Zoology, Uppsala University, Uppsala, Sweden

**MZLU** Zoological Museum–University of Lund, Sweden

**ZMUM** Zoological Museum of Moscow University

**ZIN** Zoological Institute RAN, St. Petersburg, Russia

**BL** Body length

**BW** Body width

**HT** Holotype

**PT** Paratype

## Results

### Molecular and statistical analyses

**Figure 2.**
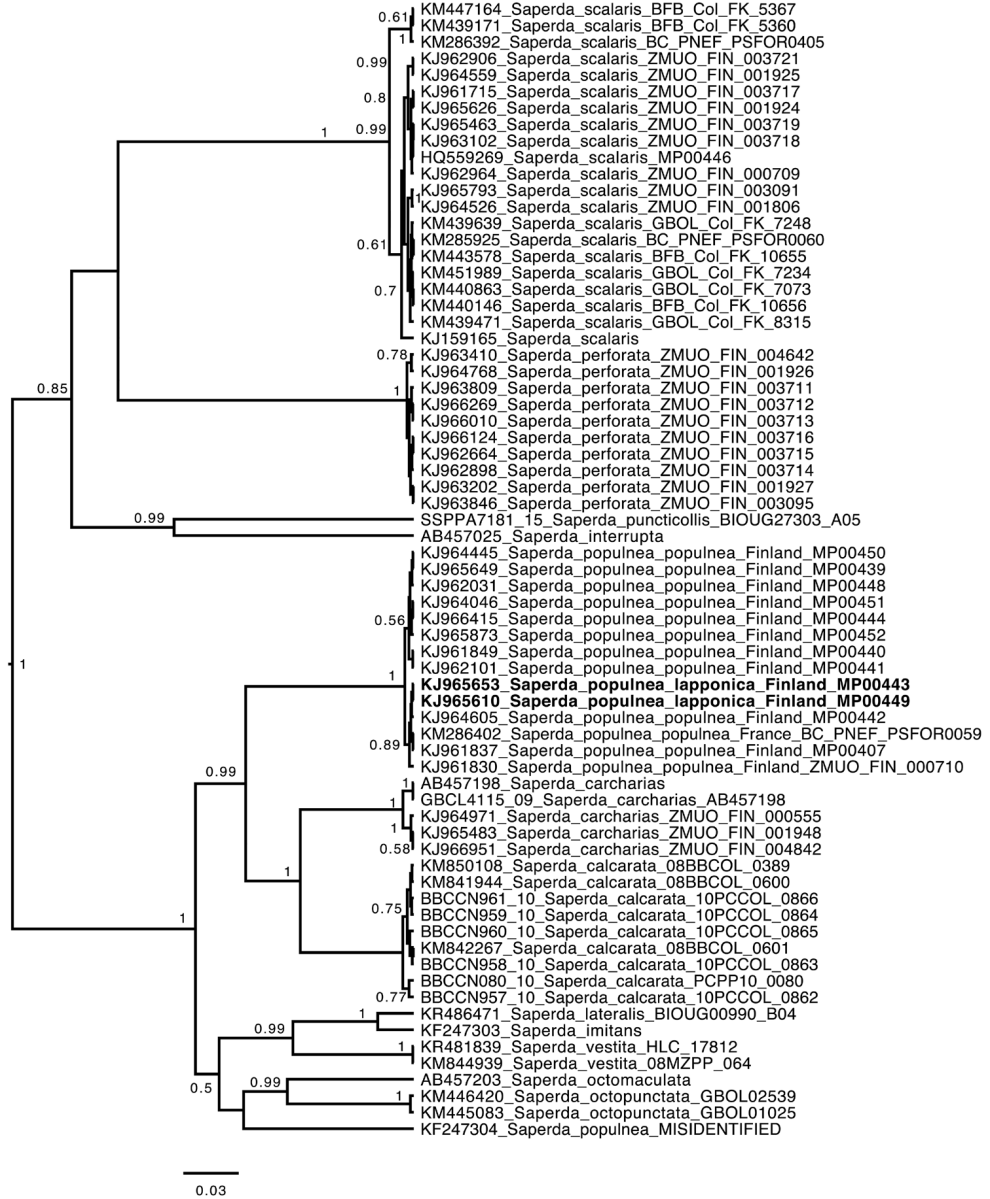
Gene tree from strict clock analysis with Beast of a 5-prime end fragment of mitochondrial cytochrome oxidase subunit I (the animal Barcoding fragment). Numbers at nodes are posterior probability values, only given for nodes >0.5. Scale bar = expected number of substitutions per site.

There are 69 published and released 5-prime end fragments of COI in Genbank and Bold of *Saperda*. The ultrametric strict clock tree from Beast recovered all *S. populnea* specimens in one monophyletic clade, apart from one released sequence from genbank (KF247304) (Fig. 2). This specimen, possibly from China, is an obvious misidentification, and must be another eastern Palearctic species of *Saperda*. The true *S. populnea* clade contained two shallow groups, one of which contained the French specimen, the two specimens from Finnish Lapponia (*S. populnea lapponica* ssp. n.) as well as three specimens from other parts of Finland (all with identical sequences apart from one bp difference in KJ964605). The two clades differed at a single position in the 407 bp long alignment. The genetic distance between specimens was 0–0.49%.

**Figure 3.**
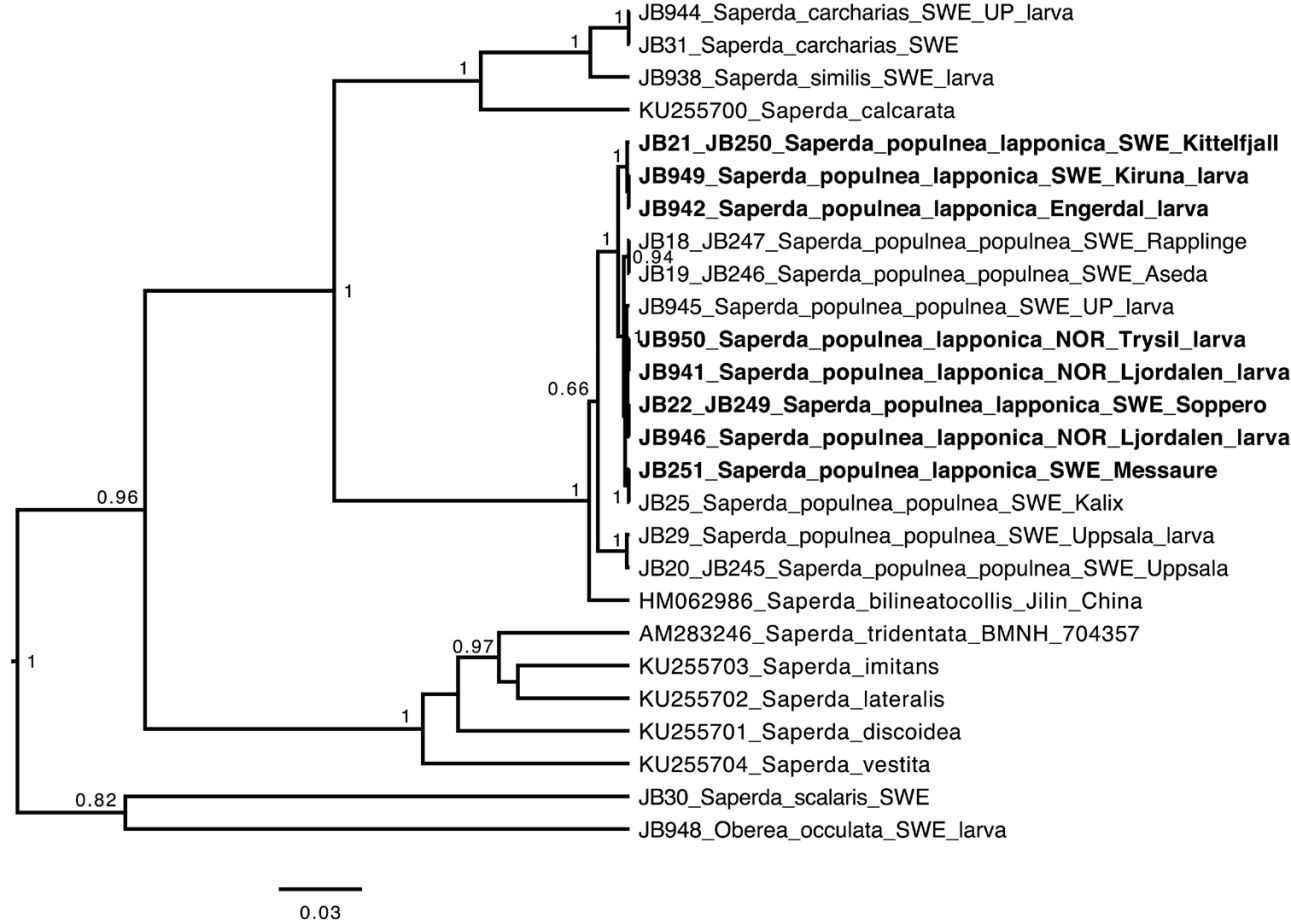
Gene tree from strict clock analysis with Beast of a 3-prime end fragment of mitochondrial cytochrome oxidase subunit I. Numbers at nodes are posterior probability values, only given for nodes >0.5. Scale bar = expected number of substitutions per site.

Amplification of the 3-prime end fragment of COI was successful for all specimens collected in the 1970s or later, but failed for all specimens from the 1960s or earlier (Tab. 1). A second independent extraction and amplification of five of the old dry-mounted specimens confirmed the sequences and assured that no cross-contamination was involved. The ultrametric strict clock tree from Beast recovered all *S. populnea* specimens in one monophyletic clade (Fig. 3). *S. populnea lapponica* ssp. n. specimens from the mountain regions of Fennoscandia and *S. populnea populnea* specimens from areas outside the mountain region were intermingled and were not reciprocally monophyletic. This included the larval specimens collected from both *Populus tremula*, and from *Salix lapponum*. A released genbank sequence submitted as *Saperda populnea* (HM062986), from Jilin province, China, came out as the most divergent and sister to remaining specimens (genetic distance: 2.09-2.60%). After receiving a photo of this specimen, we concluded that it actually refers to *Saperda bilineatocollis* Pic, 1924. There were also two moderately divergent mitochondrial clades, one of which consisted of two specimens from Uppsala, Sweden. The genetic distance of the two Uppsala specimens to the remaining Fennoscandian clade was 1.97–2.35%. The genetic distance between *S. populnea populnea* and *S. populnea lapponica* ssp. n. specimens varied between 0 to 2.35%.

The genetic distance between *S. populnea* and any of the other *Saperda* species apart from *S. bilineatocollis*, was larger, between 9.82–19.34%. The smallest interspecific distance was between *S. populnea* and *S. bilineatocollis* (2.09–2.60%) followed by *S. carcharias* and *S. similis* (2.59%). The distance between *S. populnea* and *S. bilineatocollis* (2.09–2.60%) overlaps with the distance within *S. populnea* (0–2.35%). The COI fragment of *S. similis* is the first DNA sequence released of this species.

**Figure 4.**
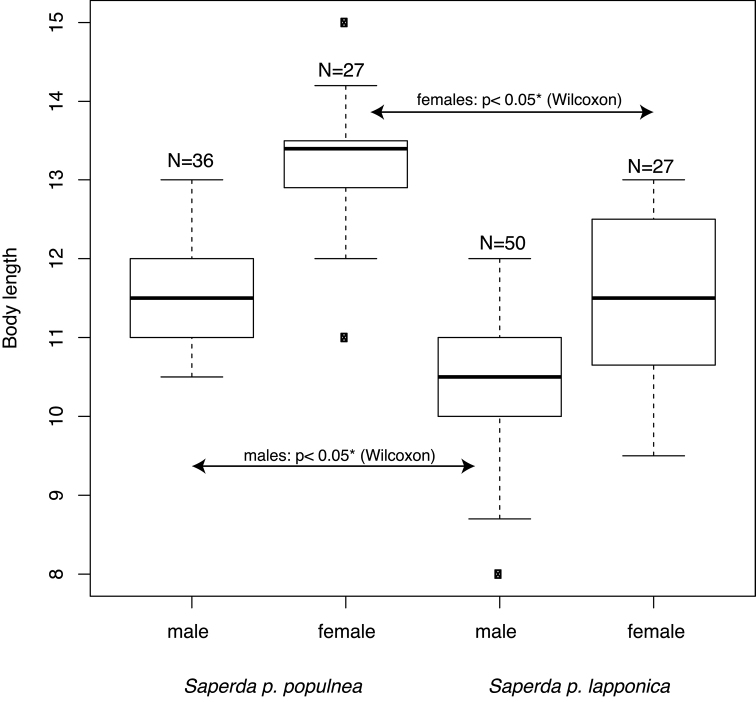
Body lengths of males and females of *Saperda populnea populnea* (Linnaeus, 1758) and *S. populnea lapponica* ssp. n. Y-axis in mm. *=significant according to a non-parametric Wilcoxon rank sum test.

The body length, among the examined specimens, was significantly smaller in *S. populnea lapponica* ssp. n. than in *S. populnea populnea* both for males (Wilcoxon p = 1.066 e-08) and for females (Wilcoxon p = 5.802 e-07) (Fig. 4). The total ranges overlapped between the examined specimens of the two subspecies (males 8–12mm vs 10.5-13.0 mm; females 9.5–13.0 mm vs 11.0–15.0 mm), but the 25-75% quartiles did not (males 10.0–11.0 mm vs 11.0–12.0 mm; females 10.7–12.5 mm vs 12.9–13.5 mm) (Fig. 4).

**Figure 5.**
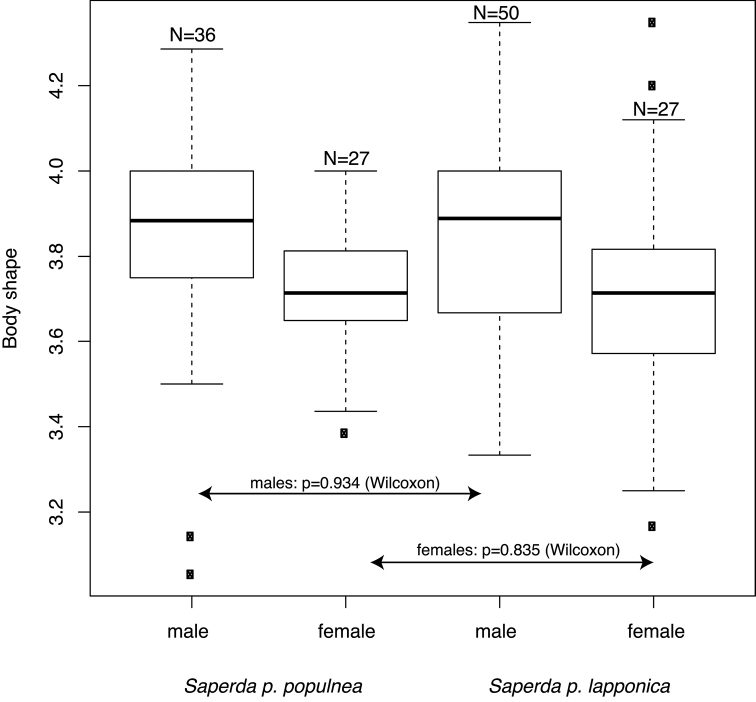
Body shape measured as the ratio of total body length to maximum body width of males and females of *Saperda populnea populnea* (Linnaeus, 1758) and *S. populnea lapponica* ssp. n. No significant difference between the subspecies of the same sex according to a non-parametric Wilcoxon rank sum test was found.

The subspecies are not diagnosable based on body length in the sense requiring 75% of individuals of subspecies A to be outside the distribution of 99% of subspecies B (Amadon 1949, Patten and Unitt 2002). The body shape measured as the ratio of body length (BL) / body width (BW) was not significantly different in either sex (Wilcoxon: males p = 0.934; females p = 0.835) (Fig. 5).

### Taxonomy

#### Genus *Saperda* Fabricius, 1775: 184

Type species. *Cerambyx carcharias* Linnaeus, 1758

*Saperda carcharias* (Linnaeus, 1758: 394).

*Cerambyx carcharias* Linnaeus, 1758 (original combination)

**Examined specimens**.

*Saperda carcharias* (Linnaeus, 1758: 394)

**Sweden**: 1 ♂ BL 24.0 mm, Uppland, Tuna Hässelby, 1980-05-05, *ex larva* from *Populus*, leg. H. Wallin, CHW; 1 ♂ BL 21.0 mm, Södermanland, Stockholm, 1993-09, leg. H. Wallin, CHW.

Saperda (Saperda) similis Laicharting, 1784: 31

**Sweden**: 1 ♂ BL 16.8 mm, Uppland, Knutby, 1995-06-05, *ex larva* from *Salix*, leg. H. Wallin, CHW; 1 ♂ BL 18.0 mm, Småland, Näsby, Bo, 1975-06-16, leg. W. Kronblad, CHW.

*Saperda scalaris scalaris* (Linnaeus, 1758: 394)

*Cerambyx scalaris* Linnaeus, 1758: 394 (original combination)

**Sweden**: 1 ♂ BL 13.8 mm, Uppland, Steninge, 1974-10-26, *ex larva* from *Quercus*, leg. H. Wallin, CHW; 1 ♂ BL 13.2 mm, Uppland, Biskops-Arnö, 1973-05-12, ex larva from *Quercus*, leg. H. Wallin, CHW.

*Saperda perforata* (Pallas, 1773: 723)

*Cerambyx perforata* Pallas, 1773: 723 (original combination)

**Sweden**: 1 ♂ BL 13.2 mm, Uppland, Uppsala, Hågadalen, 1981-06-14, leg. H. Wallin, CHW; 1 ♂ BL 13.0 mm, Uppland, Länna, 1974-06, leg. H. Wallin, CHW.

*Saperda gilanense* (Shapovalov, 2013: 139)

*Compsidia gilanense* Shapovalov, 2013: 139 (original combination)

**Iran**: **PT** ♂ BL 11.5 mm, Gassan-Kiade prov., Cefidrouda, leg. B. Ilin, 1916-04-23/24, ZIN; **PT** ♀ BL 13.7 mm, Gassan-Kiade prov., Cefidrouda, leg. B. Ilin, 1916-04-23/24, ZIN.

*Saperda quercus quercus* Charpentier, 1825: 224

*Saperda quercus* Charpentier, 1825: 224 (original combination)

**Greece**: 1 ♂ BL 14.0 mm, BW 3.5 mm, Peloponnese, Skala, Lakonia, Evrotas riv., 1994-04-24, leg. Dulik & Jeniš, CHW; 1 ♀ BL 14.0 mm, Sparti, 1991-05-31, leg. Sobota, CHW.

*Saperda bacillicornis* Pesarini & Sabbadini, 1996: 116

**China: HT** ♂ (BL not mentioned for the **HT** but overall BL is 9.1-10.3 mm), Qinghai, 40 km S Huangyuang, 1990-07-06/08, leg. Nikodym, CPS (photo examination).

*Saperda bilineatocollis* Pic, 1924: 19.

**China: HT** ♀ BL 11.0 mm, Shanghai, MNHN (photo examination). **Russia**: 1 ♀ BL 12.5mm, Kabarovsk reg. Solnetchnyi, 320m, 50°44’N, 136°39’E, 10-17.7.1991, leg. A. Shadenkov, CMD.

*Saperda innotatipennis* Pic, 1910:

**Russia: HT** ♀ BL 10.0 mm, Siberia, ex coll. Maurice Pic, MNHN (photo examination).

*Saperda messageei* Breuning, 1962: 10

**Laos: HT** ♀, Vientiane Province, Tha Ngone, 1971-07-03, ex coll. J.A. Rondon, BPBM (photo examination).

*Saperda moesta moesta* Le Conte, 1850: 234.

**Canada**: 1 ♂ BL 8.0 mm, Brittania, Hts., Ontario, 1961-07-07, on *Populus balsamifera*, leg. S.D. Hicks, NHRS; 1 ♀ BL 11.0 mm, Ross River, Y.T., 1960-06-21, leg. J.E.H. Martin, NHRS; 1 ♀ BL 9.0 mm, Quebec, Oka, 2008-06-22, leg. R. Vigneault, CHW. **USA: HT** ♀ (type no. 4213), MCZ (photo examination).

*Saperda moesta tulari* (Felt & Joutel, 1904: 70)

**USA**: 1 ♂ BL 10.0 mm, California, Stanislaus Co., Turlock, 1955-05-24, leg. R.R. Snelling, NHRS; 1 ♀ BL 10.5 mm, California, Stanislaus Co., Turlock, 1955-05-24, reared from Cottonwood, leg. R.R. Snelling, NHRS; 1 ♀ BL 9.5 mm, California, Davis, 1928-03-30, leg. F.H. Wymore, NHRS; 1 ♂ BL 8.4 mm, Nevada, leg. Morrison, NHRS no. 8147 E94; 1 ♀ BL 9.0 mm, Nevada, leg. Morrison, NHRS no. 8148 E94; 1 ♀ BL 9.8 mm, Nevada, leg. Morrison, NHRS no. 8149 E94; **HT** ♀, Tulare County, California, AMNH (photo examination).

*Saperda populnea balsamifera* (Motschulsky, 1860), **stat. n.**

*Compsidia balsamifera* Motschulsky, 1860: 151 (original combination).

**Russia**: 1 ♂ BL 9.5 mm, “less pubescent, “black” form”, S. Sachalin, Tomari, Spamberg 850 m, 1976-07-26, leg. W. Dolin, CCH; 1 ♀ BL 10.5 mm, “less pubescent black form”, Minusinsk (Siberia, Krasnojarsk region), leg. K. Ehnberg (id 772), MZH; 1 ♂ and 1 ♀ (BL 12–13 mm according to Cherepanov, 1991) “pubescent, yellow form”, Novosibirsk, 1974-07-17 on *Salix*. leg. A. Tsherepanov (photo examination), CMD; Lectotype of *Compsidia balsamifera* (probably a male, but only the elytra is preserved), ZMUM (photo examination).

#### Saperda populnea populnea

Taxon classificationAnimaliaColeopteraCerambycidae

(Linnaeus, 1758) .

**Figure 6.**
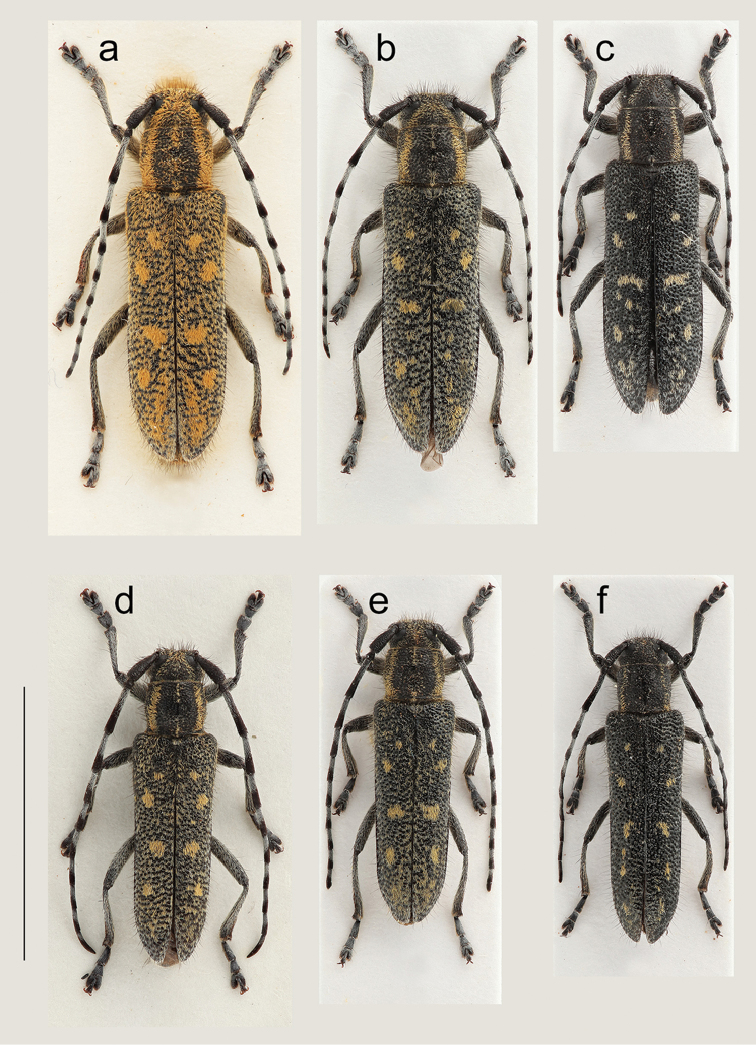
Habitus (dorsal view). **a** ♀ *Saperda populnea populnea* (Linnaeus, 1758), Knutby (Uppland), Sweden, 13.5 mm **b** ♀ *S. populnea lapponica* ssp. n., Ljørdalen, Norway, 12.5 mm **c** ♀ *S. populnea lapponica* ssp. n., Kiruna (Lappland), Sweden, 12,0 mm **d** ♂ *S. populnea populnea*, Uppsala (Uppland), Sweden, 11.5 mm **e** ♂ *S. populnea lapponica* ssp. n., Ljørdalen, Norway, 10.5 mm **f** ♂ *S. populnea lapponica* ssp. n., Kiruna (Lappland), Sweden, 10.0 mm. Scale bar 10 mm.

Figs 6a, d , 8a , 9a , 10a–b, e, g–h, k, m, o , 11a , 12c , 13

Cerambyx populneus Linnaeus, 1758: 394 (original combination). There are three males preserved at LINN available for photo examination. 1 ♂ BL 11.1 mm (LINN 8184), labelled “Populneus” on a pinned and old handwritten label and “57” (recent label added later corresponding to the number of the species in the original description by Linnaeus, type locality: “Europa” according to Linnaeus (1758), and habitat: Populus tremula according to Linnaeus (1761)); 1 ♂ BL 11.0 mm (LINN 8185), no pinned label available; 1 ♂ BL 9.2 mm, no pinned label available.
Cerambyx decempunctatus De Geer, 1775: 78 (synonymized by Breuning (1966) and Löbl and Smetana (2010)). Lectotype (here designated), ♂ BL 12.5 mm, Sweden, ex coll. De Geer, NHRS.
Leptura betulina Geoffroy, 1785: 78 (synonymised by Breuning (1966) and Löbl and Smetana (2010)).
Saperda salicis Zetterstedt, 1818: 258 (synonymised by Gyllenhal, 1827, Dejean, 1835; Breuning (1966) and Löbl and Smetana (2010)). Lectotype (here designated), ♂ BL 11.5 mm, Sweden, Skåne, Abusa, Lund (insect pin supplied with a small, square bright yellow label), 1818-08, on Salix viminalis L, leg. J.V. Zetterstedt, MZLU.
Saperda populi Duméril, 1860: 607 (synonymised by Breuning (1966) and Löbl and Smetana (2010)).
Saperda ab. bickhardti Sattler, 1918: 200 (synonymised by Breuning (1966)).
Saperda f. kavani Roubal, 1933: 133 (synonymised by Breuning (1966)).
Saperda ab. quadripunctata Podaný, 1953: 52 (synonymised by Breuning (1966)).

##### Examined specimens.

**Sweden**: 1 ♀ BL 12.5 mm, ~1818, Skåne, SE Lund, Räften Abusa etc., (insect pin supplied with a small bright yellow label), ex coll. J.V. Zetterstedt, MZLU; 1 ♀ BL 14.0 mm, ~1818, Skåne, E Lund, Björntorps säteri (insect pin supplied with small red and yellow labels), ex coll. J.V. Zetterstedt, MZLU; 1 ♂ BL 12.0 mm, ex coll. J.V. Zetterstedt, MZLU; 1 ♀ 13.0mm, ~1818, Skåne, labelled var. b., ex coll. J. V. Zetterstedt, MZLU; 1 ♀ BL 13.0 mm, ~1818, Skåne, SE Lund, Räften Abusa etc., (insect pin supplied with a small bright yellow label), ex coll. J.V. Zetterstedt, MZLU; 1 ♀ BL 13.0 mm, ~1818, ex coll. J. V. Zetterstedt, MZLU; 1 ♂ BL 11.0 mm, ~1818, Skåne, Kiviks Esperöd, (insect pin supplied with a small blue label), ex coll. J.V. Zetterstedt, MZLU; 1 ♂ BL 10.5 mm, ~1818, Skåne, E Lund, Björntorps säteri (insect pin supplied with small red and yellow labels), ex coll. J.V. Zetterstedt, MZLU; 1 ♀ BL 14.0 mm, ~1818, Skåne, E Lund, Björntorps säteri (insect pin supplied with small red and yellow labels), ex coll. J.V. Zetterstedt, MZLU; 1 ♂ BL 12.0 mm, ~1818, Skåne, E Lund, Björntorps säteri (insect pin supplied with small red and yellow labels), ex coll. J.V. Zetterstedt, MZLU; 1 ♂ BL 11.5 mm, ~1818, Skåne, labelled Cer. 10-punctata, ex coll. J.V. Zetterstedt, MZLU; 1 ♀ BL 13.5 mm, ~1818, Skåne, SE Lund, Räften Abusa etc., (insect pin supplied with a small bright yellow label), ex coll. J.V. Zetterstedt, MZLU; 1 ♀ BL 12.0 mm, ~1818, Skåne, SE Lund, Räften Abusa etc., (insect pin supplied with a small bright yellow label), ex coll. J.V. Zetterstedt, MZLU; 1 ♂ BL 11.5mm, Uppland, Uppsala, Stabby, 1991-04-19, ex larva from *Populus*, leg. H. Wallin, HW; 1 ♂ BL 12.0 mm, Norrbotten, Blåkölen, 1983-07-03, leg. S. Lundberg, NHRS; 1 ♂ BL 13.0 mm, Norrbotten, Kalix, Kosjärv, 1956-12, ex larva from *Populus tremula*, leg. S. Lundberg, NHRS; 1 ♀ BL 15.0 mm, Norrbotten, Kalix, Kosjärv, 1956-12, ex larva from *Populus tremula*, leg. S. Lundberg, NHRS; 1 ♂ BL 11.0 mm, Norrbotten, Kalix, 1994-06-30, leg. S. Lundberg, NHRS; 1 ♂ BL 12.0 mm, Småland, Åseda, ex larva from *Salix*, 1974-12-26, leg. B. Gustafsson, NHRS; 1 ♂ BL 12.2 mm, Uppland, Båtfors, 1987-06-15, leg. S. Lundberg, NHRS; 1 ♂ BL 11.5 mm, Halland, Släp, 1965-05-02, leg. L. Huggert, NHRS; 1 ♂ BL 12.0 mm, Västerbotten, Umeå, 1969-05-09, leg. L. Huggert, NHRS; 1 ♀ BL 13.0 mm, Norrbotten, Pajala, 1976-07-13, on *Populus tremula*, leg., C. Eliasson, GNM; 1 ♂ BL 10.5 mm, Bohuslän, Högås (Sund), 1947-06-16, leg. H. Arvall, NMG; 1 ♀ BL 12.0 mm, Östergötland, Omberg, 1983-06-02, on *Salix* tree, leg. S. Lundberg, NHRS; 1 ♂ BL 12.2 mm, Uppland, Uppsala, 1984-05, *ex larva* from *Salix* tree, leg. S. Lundberg, NHRS; 1 ♀ BL 13.4 mm, Öland, Räpplinge, 1976-05-03, on *Populus* tree, leg. B. Gustafsson, NHRS; 1 ♀ BL 13.5 mm, Västergötland, Amundön, 1968-12-31, ex larva, leg. L. Huggert, NHRS; 1 ♀ BL 13.5 mm, Södermanland, Nacka, Storängen, 1972-07-27, ex larva from *Populus tremula* reared 1973-05-22, leg. L. Hole, CHW; 1 ♀ BL 13.5 mm, Uppland, Knutby, 1991-05, ex larva from *Salix* tree, leg. H. Wallin, CHW; 1 ♀ BL 13.5 mm, Uppland, Knutby, Kamsgärd, 2001-07-22/29, collected in a window trap, leg. H. Wallin, CHW; 1 ♀ BL 12.8 mm, Uppland, Bladåker, 1996-07-03, on *Populus tremula*, leg. H. Wallin, CHW; 1 ♂ BL 12.0 mm, Medelpad, Sillre, leg, S. Adebratt, CUN; 1 ♂ BL 11.0 mm, Östergötland, Omberg, Stora Klint, 1983-05-25, leg. S. Adebratt, CUN; 1 ♀ BL 13.5 mm, Uppland, Knutby, 2014-11-22, reared from *Populus tremula*, leg. Å. Lindelöw, CÅL; 1 ♂ BL 12.0 mm, Uppland, Knutby, 2014-11-22, reared from *Populus tremula*, leg. Å. Lindelöw, CÅL; 1 ♀ BL 13.5 mm and 1 ♂ BL 11.3 mm, Uppland, Knivsta, 2014-10-02 (emerged 2015-02 from *Populus tremula*), leg. H. Wallin, CHW; 1 ♂ BL 11.5 mm Västerbotten, Skellefteå, 2014-05-15 (emerged 2015-02 from *Populus tremula*), CHW; 1 ♀ BL 11.0 mm, Dalarna, Los, 1924-05-25, leg. O. Sjöberg, NHRS-COLE 00007445; 1 ♀ BL 12.5 mm, Västergötland, Essunga, leg. Fogelqvist, NHRS-COLE 00007444; 1 ♀ BL 12.0 mm, Västergötland, Skövde, 1926-08-30, leg. Erlandsson, NHRS-COLE 00007431; 1 ♂ BL 12.5 mm Skåne, Sandhammaren, Bjäringeborg, 1947-06-28, leg. G. Wängsjö, NHRS-COLE 00007430; 1 ♀ BL 13.8 mm Östergötland, Norrköping, 1925-05-20, leg. G. Wängsjö, NHRS-COLE 00007424; 1 ♂ BL 11.8 mm, Blekinge, Sjöarp, 1939-06-14, leg. B. Gaunitz, NHRS-COLE 00007419; 1 ♀ BL 11.0 mm, Värmland, Filipstad, 1932-07-22, leg. K. Sidenbladh, NHRS-COLE 00007456; 1 ♀ BL 13.0 mm, Småland, Tranås, 1953-06-09, leg. L.A.H. Lindgren, NHRS-COLE 00007462; 1 ♂ BL 11.5 mm, Närke, Örebro, leg. E. Wieslander, NHRS-COLE 00007482; 1 ♀ BL 13,5 mm, Västergötland, Borås, 1938-06-13, leg. S. Åberg, NHRS; 1 ♀ BL 12.0 mm, Dalarna, Tällberg, 1958-03-25, leg. T-E. Leiler, NHRS; 1 ♂ BL 11.5 mm, ”Oel., Bhn.” (Öland ?), ex coll. Boheman), NHRS no. 8131 E94; 1 ♀ BL 14.5 mm, Stockholm, Sweden, ex coll. Hoffstein 1850-1916, NHRS-COLE 00007441; 1 ♀ BL 12.8 mm, Uppland, Uppsala, 1907, leg. O. Sjöberg, NHRS-COLE 00007442; 1 ♀ BL 13.0 Öland, leg. Ahlrot, NHRS-COLE 00007420; 1 ♀ BL 14.0 mm, Bohuslän, Ödsmål, leg. B.H. Hanson, NHRS-COLE 00007414; 1 ♀ BL 14.0 mm, Halland, Vessige, leg. Fogelqvist, NHRS-COLE 00007408; 1 ♂ BL 11.5 mm, Skåne, Hallands Väderö, 1951-06-22, leg. O. Lundblad, NHRS-COLE 00007402; 1 ♂ BL 11.0 mm, Skåne, Hallands Väderö, 1951-06-22, leg. O. Lundblad, NHRS-COLE 00007400; 1 ♀ BL 14.0 mm, Skåne, Hallands Väderö, 1951-06-22, leg. O. Lundblad, NHRS-COLE 00007401; 1 ♀ BL 13.5 mm, Öland, Ålebäck, 1947, Bg, NHRS-COLE 00007506; 1 ♀ BL 12.3 mm, Värmland, Filipstad, 1932, NHRS-COLE 00007457; 1 ♀ BL 14.5 mm, Uppland, Frösunda, 1955-12-04 (emerged from *Populus tremula*), leg. T-E. Leiler, NHRS; 1 ♂ BL 12.0 mm, Västerbotten, Umeå, 1969-05-09, leg. L. Huggert, NHRS. **Finland**: 1 ♂ BL 11.5 mm, Hammaslahti, Joensuu, 1938-06-05, leg. P. Koutkanen, NHRS; 1 ♂ BL 12.0 mm, Finland, ex coll. Schönherr., NHRS no. 8132 E94. **Norway**: 1♀ BL 12.2 mm, 23.06.1915 and 1♀ BL 10.9 mm 15.06.1915, Ø, Fr. Hald (= Fredrikshald/Halden), leg. H. K. Hanssen (ex coll. Andreas Strand), ZMUB; 1♀ BL 13.2 mm, 1♂ BL 11.3 mm, 1♀ BL 13.6 mm and 1♀ BL 13.9 mm, Ø, Fr. Hald (= Fredrikshald/ Halden), 1905, leg. Lyche (ex coll. Ing. Tambs-Lyche), ZMUB (e c G1994); 1♀ BL 12.8 mm, Ø, Fr. Stad (= Fredrikstad), 20.05.1895 (ex coll. E. Sandberg) ZMUB (e c G1994); 1♂ BL 10.9 mm, Ø, Aaldenborgilen (= Oldenborgila), Fr.stad (= Fredrikstad/Halden), 29.05.1895, leg. A. Wollebæk, ZMUB (e c M2951); 1 ♂ BL 10.5 mm, Ø, Aaldenborgilen (= Oldenborgila), Fr.stad (= Fredrikstad), 1895-05-26, leg. A. Wollebæk, NHMO; 1 ♂ BL 11.0 mm, Ø, Aaldenborgilen (= Oldenborgila), Fr.stad (= Fredrikstad), 1895-05-26, leg. A. Wollebæk, NHMO; 1♀ BL 12.5 mm, Ø, Romskogen (= Rømskog), Leg. Holmboe according to Andreas Strand, (ex coll. Andreas Strand) ZMUB; 1♀ BL 12.3 mm, Ø, Romskog (= Rømskog), Leg. Holmboe according to Andreas Strand, (ex coll. Andreas Strand), ZMUB; 1 ♂ BL 10.5 mm, AK, Kristiania (= Oslo), leg. Siebeke, NHMO; 1♀ BL 13.6 mm, AK, Bygdø (in Oslo), 12. 07.1907, leg. Lyche (ex coll. Ing. Tambs-Lyche) ZMUB (e c G1994); 1 ♂ BL 11.3 mm, EIS 37, AK, Sørum, Sørliløkka, Dammyra, 1991-06-17, leg. O. Sørlibråten, COS; 1 ♂ BL 10.5 mm, AK, Oslo, Brannfjell, 2007-06-05, inside gall on *Populus tremula*, leg. A. E. Laugsand, CAEL; 1 ♀ BL 12.0 mm, AK, Oslo, Brannfjell, 2007-06-05, inside gall on *Populus tremula*, leg. A. E. Laugsand, CAEL; 1♀ BL 13.4 mm, AK, V. Aker, Oslo, 1907, Leg. Lyche (Ex coll. Ing. Tambs-Lyche) ZMUB (e c G 1994); 1♂ BL 11.7 mm, 1♂ BL 12.3 mm and 1♀ BL 12.7 mm, AK, Bækkelag (in Oslo), before 1892, leg. N. G. Moe?, ZMUB (e c G1995); 1♂ BL 11.1 mm and 1♀ BL 12.0 mm, AK, Kristiania (= Oslo), before 1884, Leg. Esmark, ZMUB (e c M2950); 1♀ 10.5 mm and 1♂ BL 11.4 mm, AK, Kristiania (= Oslo), leg. Warloe?, (ex coll. Andreas Strand) ZMUB; 1♀ BL 13.7 mm, AK, Brønnøya, Asker, 15.06.1961, leg. Andreas Strand, (ex coll. Andreas Strand) ZMUB; 1♀ BL 12.5 mm, AK, Brønnøya, Asker, 16.06.1934, leg. Andreas Strand, (ex coll. Andreas Strand) ZMUB; 1 ♀ BL 12.5 mm, AK, Drøbak, before 1939, leg. Warloe, ZMUB (e c M2952); 1♂ BL 10.3 mm, AK, Drøbak, 03.06.1895, leg. Warloe, (ex coll. Andreas Strand) ZMUB; 1♀ BL 12.8 mm, AK, Drøbak, 06.08.1895, leg. Warloe, (ex coll. Andreas Strand) ZMUB; 1 ♀ BL 14.1 mm, AK, Drøbak, 01.06.1895, leg. Warloe, ZMUB (e c M2952); 1 ♂ BL 11.2 mm, AK, Drøbak, 03.06.1895, leg. Warloe, (ex coll. Andreas Strand) ZMUB; 1 ♂ BL 11.0 mm, AK, Drøbak, 1891-06-25, leg. Warloe, NHMO; 1 ♂ BL 11.5 mm, AK, Drøbak, 1891-06-25, leg. Warloe, NHMO; 1 ♀ BL 14.3 mm and 1♂ BL 11.3 mm, VE, Nøterø (= Nøtterøy), 20.VI.1921, Leg. H. Tambs-Lyche, (ex coll. Ing. Tambs-Lyche) ZMUB (e c G1994); 1♀ BL 12.3 mm, VE, Tjømø (= Tjøme), 08.07.1909, Leg. Lyche, (ex coll. Ing. Tambs-Lyche) ZMUB; 1♀ BL 11.0 mm, VE, Kjære, Tjøme, 09.06.1965, on osp (=*Populus tremula*) Leg. A. Fjellberg, ZMUB; 1 ♂ BL 11.0 mm, VE, Sandefjord, 1978-07-20, on *Populus tremula*, leg. A. Vik, (coll. NIBIO) NHMO; 1♂ BL 11.6 mm, AAY, Risør, 26.05.1918, leg. Warloe, (Ex coll. Andreas Strand) ZMUB; 1♀ BL 13.6 mm, RY, Fotlandsvatn, Eigersund, 29.05.1973, A. Fjeldså, ZMUB. **Germany**: 1 ♂ S. populnea var. quadripunctata Podaný BL 11.2 mm, Westfalen, 1966-06-09, leg. K. W. Stockmann (id 1377), MZH; 1 ♀ BL 12.3 mm, Märzat, 1920-05-27, NHRS no. 8136 E94; 1 ♀ BL 13.5 mm, Boruss. (= Prussia), Mewes, NHRS no. 8137 E94; 1 ♀ BL 13.5 mm, Boruss. (= Prussia), Mewes, NHRS no. 8138 E94; 1 ♂ BL 12.0 mm, Boruss. (= Prussia), Mewes, NHRS no. 8141 E94; 1 ♂ BL 11.0 mm, Heidelberg, det. E. F. Gilmour, NHRS no. 8140 E94. **Austria**: 1 ♂ BL 10.5 mm, “Austria”, leg. Ferrari, NHRS no. 8133 E94; 1 ♂ BL 11.0 mm, “Austria”, leg. Ferrari, NHRS no. 8134 E94; 1♂ BL 11.3 mm, Umbegung von Wien, leg. ?, (ex coll. Andreas Strand) ZMUB. **France**: 1 ♀ BL 13.6 mm, Gallia Meridionalis (=South France), Tarnier, NHRS no. 8139 E94; 1 ♂ BL 11.7 mm, La Roquebrussanne (Var), 2008-06, local collector, CHW; 1 ♀ BL 13.5mm, La Roquebrussanne (Var), 2008-06, local collector, CHW; 1 ♂ BL 10.5 mm, Aramon, Var, 2015-05-12/15, CHW; 1 ♀ BL 11.0 mm, Aramon, Var, 2015-05-12/15, CHW. **Switzerland**: 1 ♀ BL 13.5 mm, 1 ♀ BL 12.5 mm, 1 ♂ BL 11.0 mm and 1 ♂ BL 10.1 mm, Münstertal, Santa Maria, 1400 m, 1953-06-19/22, leg. Lindberg, MZH. **Czech Republic**: 1 ♀ BL 12.5 mm, Zbraslav (Prag), 1990-05, leg. Rejzek, CHW. **Czech Republic or Poland**: 1 ♀ BL 13.0 mm, Märztdorf, leg. Weisse, NHRS no. 8135 E94; 1 ♂ BL 11.0 mm, Märztdorf, leg. Weisse, NHRS no. 8135 E94. **Kazakhstan**: 1 ♀ BL 11.0 mm, S. Kazachstan, Alma-Ata, 2000–2300 m, 1977-06-20–07-05, leg. V. Dolin, det. M. Danilevsky 2003, CCH. **Indonesia**[?]: 1 ♀ BL 12.5 mm, “Java”, ex coll. Schönherr, NHRS no. 8142 E94.

##### Additional material examined.

The following specimens are available through Boldsystems Public Data Portal and MZH for photo examination and include: **Finland**: 1 ♂ COLFA145-10, Northern Ostrobothnia (= Österbotten), Oulu, *ex larva* April 2005, leg. Mikko Pentinsaari, Marko Mutanen, id MP00407, ZMUO; 1 ♂ COLFA177-10, SW Finland, Eurajoki (N. Rauma), *ex larva* 1996, *Salix phylicifolia*, leg. Juhani Itaemies, id MP00439, ZMUO; 1 ♀ COLFA178-10, Nylandia, Uusimaa, Espoo, *ex larva* 1997, *Populus tremula*, leg. Juhani Itaemies, id MP00440, ZMUO; 1 ♀ COLFA179-10, Nylandia, Uusimaa, Espoo, *ex larva* 1996, *Populus tremula*, leg. Juhani Itaemies, id MP00441, ZMUO; 1 ♀ COLFA180-10, SW Finland, Satakunta, Rauma, *ex larva* 1982, *Populus tremula*, leg. Juhani Itaemies, id MP00442, ZMUO; 1 ♂ COLFA182-10, SW Finland, Mynaemaeki, *ex larva* 1996, *Salix caprea*, leg. Juhani Itaemies, id MP00444, ZMUO; 1 ♀ COLFA186-10, SW Finland, Lappi (SE Rauma), *ex larva* 1992, *Salix caprea*, leg. Juhani Itaemies, id MP00448, ZMUO; 1 ♂ COLFA188-10, Uusimaa, Nylandia, Kirkkonummi (= SE Lohja), *ex larva* 2003, leg. Erkki Laasonen, id MP00450, ZMUO; 1 ♂ COLFA189-10, Satakunta, SW Rauma, *ex larva* 1982, *Populus tremula*, leg. Juhani Itaemies, id MP00451, ZMUO; 1 ♀ COLFA190-10, Satakunta, Rauma, *ex larva* 1991, *Salix phylicifolia*, leg. Juhani Itaemies, id MP00452, ZMUO; 1 ♂ COLFA575-12, Nylandia, Uusimaa, Vartiokylae (= SE Vantaa), 2008-06-27, leg. Sami Haapala, id MP00452, ZMUO; 1 ♀ Porvoo, 31.12.1965 (ex larva), leg. H. Valtari, MZH; 1 ♀ Turku (= Åbo), 2.2.1971 (ex larva), leg. E. Linnaluoto, MZH; 1 ♀ Ruokolahti, Haloniemi, 22.6.1948, leg. W. Hellén, MZH; 1 ♀ Ruokolahti, Rasila, Patjasuo, 22.6.1948, collector unknown, MZH; 1 ♀ Kuhmoinen, collection date not available, leg. M. Pohjola, MZH; 1 ♀ Kirkkonummi, 4.6.1919, leg. Håkan Lindberg, MZH; 1 ♀ Borgå, Seitlax, 18.6.1920, leg. Thuneberg, MZH; 1 ♀ Kouvola, Voikkaa, date not available, leg. Paulamo, MZH; 1 ♀ Kangasala (= E. Tampere), collection date not available, leg. Grönblom, MZH; 1 ♀ Hämeenlinna, Vanaja, 31.12.1957 (ex larva), leg. Valkeila, MZH; 1 ♀ Mikkeli, 30.1.2001 (ex larva), leg. M. Koponen, MZH; 1 ♀ Kankaanpää, collection date not available, leg. M. Pohjola, MZH; 1 ♀ Kokemäki, Kauvatsa, 2.7.1934, leg. R. Elfving, MZH; 1 ♀ Parikkala, Laurila, 16-27.6.1940, leg. S. Hellén, MZH; 1 ♀ Kouvola, Kuusankoski, 31.12.1986 (ex larva), leg. J. Jantunen, MZH; 1 ♂ Lapua, 31.12.1971 (ex larva), leg. R. Järvenpää, MZH; 1 ♂ Keuruu, 31.12.1971 (ex larva), leg. R. Järvenpää, MZH; 1 ♀ Jyväskylä, 30.01.1975 (ex larva), leg. J. Jalava, MZH; 1 ♀ Pieksämäki, 30.01.1975 (ex larva), leg. J. Jalava, MZH; 1 ♂ Kuopio, collection date not available, leg. Kurkiharju, MZH; 1 ♀ Kitee, 31.12.1938 (ex larva), leg. J. Kaisila, MZH; 1 ♀ Juuka, 2.7.1949, leg. Wegelius, MZH; 1 ♀ Joensuu, collection date not available, J. Carpelan, MZH; 1 ♀ Hangö (= Hankö), Lappvik, 16.6.2009, leg. H. Silfverberg, MZH; 1 ♀ Parainen, Nauvo, 16.6.1960, leg. A. Nordman, MZH; 1 ♂ Loppi, 30.6.1943, leg. A. Saarinen, MZH. **Russia**: 1 ♂ Republic of Karelia, Viipuri (= Vyborg), 18.6.1920, leg. Thuneberg, MZH; 1 ♀ Leningrad (= St. Petersburg) Oblast, Kuolemajärvi (Pionerskoye), 10.6.1917, leg. M. Ivaschinzeff, MZH; 1 ♀ Republic of Karelia, Impilahti (= Impilaks), collection date not available, leg. Forsius, MZH.

##### Redescription.

A medium-sized and subcylindrical species with body length 9.0–15.0 mm according to e.g. Freude et al. (1966), Bilý and Mehl (1989), Bense (1995) and Ehnström and Holmer (2007). Measurements from the present study; females: BL 11.0–15.0 mm and males: BL 10.5–13.0 mm. Body 3.1 times longer than wide in females and 3.3 times longer than wide in males (Fig. 6a, d). Integument black, the compressed pubescence is orange-brown, with numerous long, erected dark brown hairs. The orange-brown pubescence relatively dense in males and from dense to very dense in females, resulting in females being more orange-brown, and males grayish to orange-brown (Fig. 6a, d). The orange-brown pubescence is extended laterally in females, especially on pronotum, anterior part of elytra and abdomen (Fig. 8a).

**Head in females.** Frons convex and broader than long (about 4.7 times broader than the width of one eye lobe), eyes with lower eye lobes longer than broad and, as long as, or slightly longer than gena below. Head with frons more or less “square-formed” in many female specimens, genae straight and acutely narrowing towards mouthparts (Fig. 9a), frons densely covered with orange-brown pubescence and numerous dark brown, long and erected hairs. Genae posteriorly with long fringes of orange-brown hairs. The area between antennal segments is shallowly impressed. **Head in males.** Frons convex and broader than long (about 4.5 times broader than the width of one eye lobe), eyes with lower eye lobes longer than broad and 2-3 times longer than the short gena below. Head with frons rounded, genae straight and acutely narrowing towards mouthparts, frons densely covered with whitish and orange-brown pubescence and numerous dark brown, long and erected hairs. Genae posteriorly with long fringes of orange-brown hairs. The area between antennal segments is shallowly impressed. **Mouthparts.** Frontoclypeal margin with a fringe of relatively long orange-brown pubescence and long, orange brown, suberect hairs. Clypeus glabrous except at base. Labrum with appressed orange-brown pubescence and numerous long, suberect, orange-brown hairs. **Antennae.** Relatively slender, about as long as body in males (Fig. 6d), shorter in females (Fig. 6a). The length of antennae varies in males from antennae extending beyond apices by one antennomere to shorter than elytra by three antennomeres. The length of antennae varies less in females with antennae extending beyond the middle of elytra by 3–5 antennomeres. Antennae from third segment with annulation. Scape slender and coarsely punctured with a combination of large and small shallow punctures and long black hairs, subconical, third segment longer than first and fourth. Annulation on antennal segments greyish and covering about ¾ of the anterior part of each antennal segment. **Thorax.** Pronotum subcylindrical, slightly broader than long, lacking lateral spines. Pronotal disk convex, weak median line often with a glabrous and shining area medially, base shallowly impressed, coarse punctures except medially, densely covered with long erect and brown hairs, two broad lateral orange-brown stripes with a weak median line interrupted medially, prosternum densely pubescent with orange-brown hairs. **Elytra.** 2.5–2.9 times longer than broad in females and 2.7–3.0 times longer than broad in males. No carinae present. Parallel and weakly narrowing towards apices, apices narrowing and rounded, punctures coarse, deep, contiguous towards humeri and apices and confluent medially (especially in males where confluent punctures form short and weakly raised ridges transversally on each elytron), pubescence dense to very dense. There are normally eight distinct and large, orange-brown spots on elytra (apart from an irregular patch of orange-brown pubescence often occurring towards apices). The eight rounded spots are arranged in pairs with the first and third near the suture; each spot in the third pair often elongated transversally or even divided into two spots each; spots in the fourth pair sometimes slightly elongated longitudinally. One or more pairs of spots may be obsolete or rarely missing (particularly in old worn specimens). The remaining part of elytra is covered with scattered orange-brown pubescence and numerous long brown hairs. **Scutellum.** “U-shaped” and covered with orange-brown hairs, the hairs are mostly concentrated to the middle of scutellum. **Hind wing.** About 12.0 mm long in females and about 10.0 mm long in males (Fig. 11a). Covered with a weak smoky tint. Several veins are broken with apical portions not connected to basal portions. MP3 (rudimentary), MP4 and AA vein distinct although broken. Radial cell very strong and complete (Fig. 11a). **Legs.** Relatively short, densely covered with a fine whitish pubescence including tarsi; tarsal claws lacking a process. **Venter.** Densely covered with orange-brown pubescence in both sexes, prosternal process narrow and flattened anteriorly. Mesosternum and abdominal ventrites are densely covered with orange-brown pubescence and numerous yellowish and long, erected hairs. Posterior margin of sternite VII mostly rounded but sometimes weakly notched medially. **Male terminalia.** Aedeagus 2.1–2.5 mm long, weakly curved towards apex and compressed dorso-ventrally (Figs 10a–b, 9e), dorsal surface smooth and shining with apical part strongly to moderately narrowed towards apex (Fig. 10e). Tegmen with parameres: 2.2–2.7 mm long with tegmen mostly twisted dorso-ventrally (Fig. 10k). Parameres acutely narrowing towards apex, with dorsal surface densely covered with punctures and suberected setae. The inner margins mostly well separated and diverging towards apices (Fig. 10h) but sometimes projecting inwards (Fig. 10g). Tergite VIII 0.7–1.0 mm long relatively large and rounded with a posterior margin concave in the middle and densely covered with dense white pubescence and numerous long brown hairs (Fig. 10o). Sclerite inside internal sac: 1.8–2.2 mm long consisting of three parallel “shaft-like” structures of which the apical end (top) is elongated and posterior end often extended and narrowing towards posterior end (Fig. 10m). The colour of male genitalia is brownish. **Female terminalia.** Tignum almost straight, 6.4–8.5 mm long (width 0.1–0.2 mm at the widest point apically). Tergite VIII posterior margin (width: 1.0 mm) with a few brown hairs. The colour is brown. Spermathecal capsule strongly sclerotised, yellowish, round and supplied with a short shaft, diameter: 0.5 mm.

**Figure 7.**
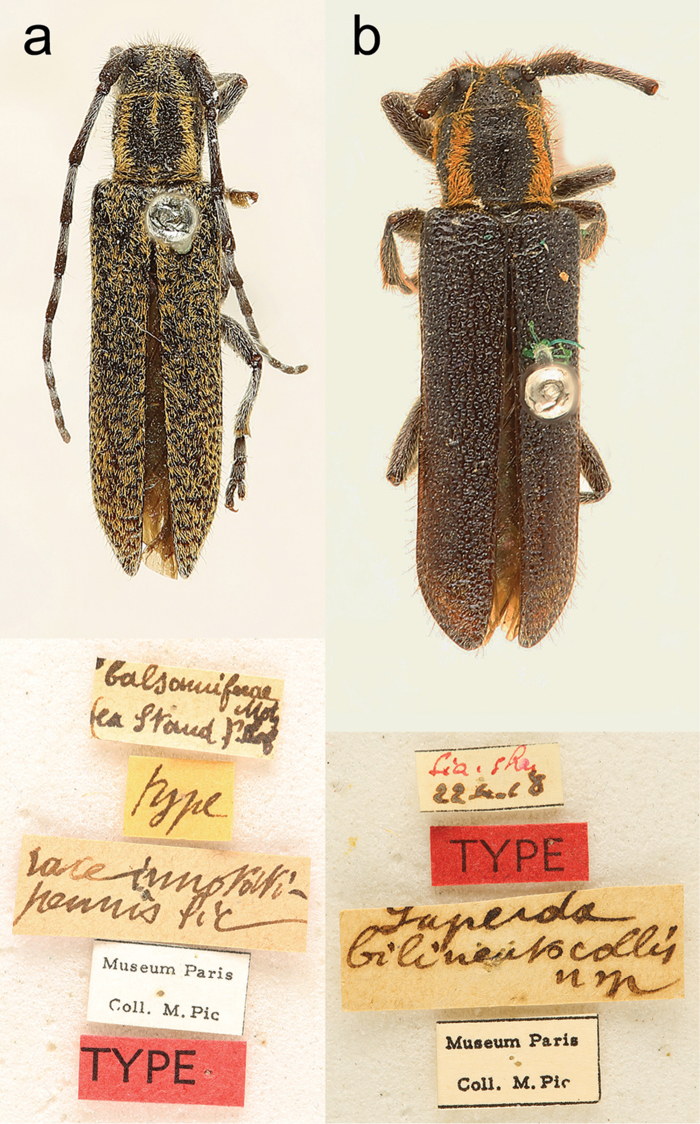
Habitus (dorsal view). **a** HT ♂ *S. innotatipennis* Pic, 1910, 10.0 mm (photo: **a** Taghavian, MNHN) **b** HT ♀ *S. bilineatocollis* Pic, 1924, 11.0 mm (photo: **b**. Taghavian, MNHN).

##### Remarks.

Morphological characteristics of *S. populnea populnea* are based on type specimens preserved at LINN (males). The host tree was claimed by Linnaeus (1761) to be *Populus tremula*. *S. populnea populnea* is a highly variable species, the most common form in Europe having extensive orange-brown pubescence on pronotum and elytra and four distinct pairs of orange-brown spots on elytra. The orange-brown pubescence on elytra (apart from the four pairs of spots) is often reduced especially in females, as a result of variation, but also in old worn specimens. The characters presented herein are therefore mainly based on newly hatched and fully sclerotised specimens reared from *Populus tremula*.

##### Distribution.

*S. populnea* is the most widespread and variable species within the genus, with populations occurring in almost the entire Palaearctic region from the British Isles in the west to Far East of Russia and China in the east (Löbl and Smetana 2010). *S. populnea populnea* is common in Fennoscandia, although less frequently found in Norway in the past. It was recorded from Northern Norway (Strand 1946, Bily and Mehl 1989, Ehnström and Holmer 2007). We have not seen any of these specimen(s) from Northern or Western Norway and consequently, we do not know the identity of the subspecies. Distribution patterns over the past 200 years in Sweden show stable populations in the southern provinces, with only a few records in the Northern provinces, mainly along the coast (Lindhe et al. 2010). Most records of the examined specimens of *S. populnea populnea* from Fennoscandia are from coastal areas in southern Norway and Finland and numerous inland records from southern Sweden and Finland. Only a few specimens have been recorded in inland, northern Sweden (Fig. 13).

##### Biology.

Females form a “U-shaped mark” in the bark of *Populus tremula*, on stems and branches 1–2 cm in diameter, forming a lid under which an egg is deposited. Usually, a single larva is tunnelling in the centre of the branch of living aspens, where the host tree responds by forming a more or less distinct gall (Ehnström and Axelsson 2002, Lindhe et al. 2010). An attack by female larvae often results in larger galls than those initiated by male larvae (Fig. 12c). Normally, only scattered attacks can be found in the same habitat with only one or two galls on the same stem or twig. Damages caused by mass attack of *S. populnea populnea* have been observed in many European countries (e. g. Schwenke 1974) as well as in Asia (e.g. Cherpanov 1991). In Sweden, *Populus* plantations have been severely damaged (Ehnström and Axelsson 2002). Today the species has become less abundant in Sweden. Only few and scattered records are known from northern Sweden (Lindhe et al. 2010). The development takes 2 years. The biology and larval morphology of *S. populnea* has been dealt with by many authors (e.g. Duffy 1953, Demelt 1966, Schwenke 1974, Cherepanov 1991, Švácha 2001, Ehnström and Axelsson 2002). We have included specimens of *S. populnea populnea* from all Fennoscandian countries and as many northern records as possible (Fig. 13).

##### Host tree.

The preferred host tree is *Populus tremula* as a number of authors have claimed (Tab. 2). A total of 16 other *Populus* species and hybrids are attacked in Europe and Asia (Tab. 2). Many species of the genus *Salix* are also reported to be attacked (Tab. 2). Amongst the specimens included in the present study, the majority was reared from *Populus tremula*, while only a few specimens were reared from *Salix caprea* L. and *S. cinerea* L. *S. populnea populnea* is also known to attack living branches and narrow stems of other trees and shrubs in Europe e.g. *Corylus* spp. (Bense 1995) and *Betula* sp. (Vives 2000, Sama 2002), although *Betula* spp. has not been recorded as a host tree in any of the Nordic countries. We, therefore, question the validity of *Betula* sp. as a host tree. Hua (2002) and CABI (2012) mentioned *Quercus glauca* Thunb. (apart from five species of *Populus*) as a host tree in China. *S. populnea populnea* has also been reared from stems of coppiced hedgerow ash (*Fraxinus excelsior* L.) in the UK (Allen 1979) (Tab. 2).

**Table 2.** Host tree species of *Saperda populnea populnea* (Linnaeus, 1758) based on data from literature.

Host tree species	Reference
*Populus tremulae* L.	Aurivillius 1917, Strand 1946, Horion 1974, Schwenke 1974, Bílý and Mehl 1989, Burakowski et al. 1989, Cherepanov 1991, Bense 1995, Slama 1998, Vives 2000, Ehnström and Axelsson 2002, Heliövaara et al. 2004, Böhme 2005, Ehnström and Holmer 2007, Lindhe et al. 2010, Berger 2012, CABI 2012
*Populus* spp.	Demelt 1966, Schwenke 1974, Horion 1974, Burakowski et al. 1989, Cherepanov 1991 Bílý and Mehl 1989, Bense 1995, Slama 1998, Sama 2002, Ehnström and Axelsson 2002, Ehnström and Holmer 2007
*P. nigra* L.	Vives 2000, Berger 2012, CABI 2012
P. nigra var. thevestina	Hua et al. 2009
P. nigra var. italica	CABI 2012
*P. alba* L.	Vives 2000, Hua et al. 2009, Berger 2012, Cabi 2012
*P. canadensis* Moench	Hua 2002, Hua et al. 2009, CABI 2012
*P. cahtayana* Redh.	Hua 2002, Hua et al. 2009
*P. simonii* Carr.	Hua 2002, Hua et al. 2009, CABI 2012
*P. pseudosimonii* Kitag.	CABI 2012
*P. davidiana* Dode.	Hua 2002, Hua et al. 2009
*P. tomentosa* Carr.	Hua et al. 2009, CABI 2012
*P. xiaozhuanica* W.Y.Hsu & Liang	CABI 2012
*P. nigra* x *P. deltoides* (Canadian poplar)	Schwenke 1974
*Populus* x *wettsteinii* (Hybrid aspen)	Ehnström and Holmer 2007
*Populus* x *euramericana*	CABI 2012
*P. tremula* x *P. tremuloides*	Löyttyniemi 1972
*P. alba* x glandulosa	Park and Kim 1986, CABI 2012
*Salix* spp.	Demelt 1966, Cherepanov 1991, Strand 1946, Horion 1974, Schwenke 1974, Burakowski et al. 1989, Bense 1995, Vives 2000, Sama 2002, Ehnström and Axelsson 2002, Heliövaara et al. 2004, Ehnström and Holmer 2007, Lindhe et al. 2010,
*S. caprea* L.	Burakowski et al. 1989, Slama 1998, Heliövaara et al. 2004, Hua et al. 2009, Berger 2012
*S. phylicifolia* L.	Heliövaara et al. 2004
*S. alba* L.	Hua et al. 2009
*S. cinerea* L.	Burakowski et al. 1989
*S. viminalis* L.	Burakowski et al. 1989, Hua et al. 2009
*Fraxinus excelsior* L.	Allen 1979, CABI 2012
*Bischofia javanica* Blume	CABI 2012
*Corylus* sp.	Sama 1988, Bense 1995
*Betula* sp.	Vives 2000, Sama 2002
*Quercus glauca* Thunb.	Hua 2002, CABI 2012

#### Saperda populnea lapponica ssp. n.

Taxon classificationAnimaliaColeopteraCerambycidae

http://zoobank.org/85C74E08-E401-48AA-8463-FF5AFC1D9835

Figs 1 , 6b–c, e–f , 8b , 9b , 10c–d, f, i–j, l, n, p–q , 11b–c , 12a–b , 13

##### Type material.

**Holotype**: ♂ NHRS (id NHRS-JLKB0000027179), BL 11.0mm, BW 3.0mm, from Sweden, Lappland, Lule lappmark, 2 km SE Kiruna, elev. 500 m, ”Aptasvaara”, reared from *Salix lapponum* 2014-07-09 (emerged 2015-02), leg. H. Wallin. **Paratypes: Sweden**: 1 ♀ BL 11.0 mm, same data as holotype, NHRS; 1 ♀ BL 10.0 mm, same data as holotype, CHW; 1 ♀ BL 9.5 mm and 1 ♂ BL 11.0 mm, same data as holotype, CHW; 1 ♀ BL 11.5 mm and 1 ♂ BL 10.5 mm, Sweden, Lappland, Lule lappmark, 20 km NW Kiruna, ”Gallugas”, reared from *Salix lapponum* 2015-06-11 (emerged 2015-06-24), leg. H. Wallin, CHW; 1 ♂ BL 11.0 mm, 1 ♂ BL 10.0 mm, 1 ♂ BL 9.5 mm and 1 ♀ BL 11.7 mm, Jämtland, Ånn (5 km W. Tångböle), Åre, reared from *Salix lapponum* 2016-09-12/13 (emerged 2017-01), leg. H. Wallin, CHW. 1 ♂ BL 12.0 mm, Lappland, Lule lappmark, Messaure, 1971-07-14/21, window trap, leg. T. Mûller, NHRS; 1 ♂ BL 10.5 mm, Lappland, Lule lappmark, Litnok, 1967-07-21, leg. S. Lundberg, NHRS; 1 ♂ BL 11.0 mm, Lappland, Torne lappmark, Sappisatsi, N. Vittangi, 1966-07-04, leg. S. Lundberg, NHRS; 1 ♂ BL 11.0 mm, Lappland, Torne lappmark, Soppero, 1968-06-15, ex larva reared from *Salix lapponum*, leg. S. Lundberg, NHRS; 1 ♂ BL 10.0 mm and 1 ♀ BL 10.5 mm, Lappland, Torne lappmark, Soppero, 1980-06-30, leg. S. Lundberg, NHRS; 2 ♂♂ BL 10.0 mm and 1 ♀ BL 9.5 mm, Lappland, Torne lappmark, Siltimuotka, Soppero, 1948-06-28, leg. N. Höglund, NHRS; 1 ♂ BL 11.5 mm, Lappland, Åsele lappmark, Kittelfjäll, 1972-06-28, leg. T-E. Leiler, NHRS; 1 ♀ BL 11.2 mm and 1 ♀ BL 10.5 mm, Lappland, Torne lappmark, Kiruna, ex larva from *Salix lapponum*, leg., E.v. Mentzer, CBE; 1 ♂ BL 11.0 mm, Jämtland, Tångböle, Åre, 1964-07-07 (locality J23 in a survey), leg. Waldén, Enckell & Hagberg, NMG; 1 ♂ BL 10.5 mm and 1 ♀ BL 13.0 mm, Lappland, Torne lappmark, Kiruna, Aptasvaara, 1976-07-10, on *Salix lapponum*, leg., C. Eliasson, GNM; 1 ♂ BL 10.3 mm, 1 ♂ BL 10.5 mm and 1 ♀ BL 12.4 mm, Lappland, Lycksele lappmark, Tärnaby, Juksjaur, 2013-06-30, on *Salix lapponum*, leg. R. Petterson, CRP; 1 ♂ BL 11.0 mm, Jämtland, Järvsand, 1986-06-19, leg. R. Petterson, CRP; 1 ♀ BL 12.0 mm, labelled “Zetterstedt”, ex coll. Gyllenhal, UUZM; 1 ♂ BL 10.0 mm, labelled “Zetterstedt”, ex coll. Gyllenhal, UUZM; 1 ♀ BL 10.0 mm, 1 ♂ BL 8.0 mm, 1 ♂ BL 10.2 mm, 1 ♂ BL 9.0 mm, Dalarna, Idre, 2014-06-26, reared from *Salix lapponum*, leg. Å. Lindelöw, CÅL; 1 ♀ BL 12.0 mm, 1 ♀ BL 11.3 mm, 1 ♂ BL 11.0 mm, 2 ♂♂ BL 10.0 mm, 2 ♂♂ BL 10.5 mm Lappland, Lule lappmark, 2 km SE Kiruna, elev. 500 m,”Aptasvaara”, beaten from *Salix lapponum* 2014-07-09, leg. H. Wallin, CHW; 1 ♀ BL 12.0 mm, 1 ♀ BL 11.0 mm, 1 ♂ BL 9.5 mm, 1 ♂ BL 10.0 mm, Lappland, Lule lappmark, 2 km SE Kiruna, elev. 500 m, ”Aptasvaara”, reared from *Salix lapponum* 2014-07-09 (emerged 2015-02), leg. H. Wallin, CHW; 1 ♂ BL 11.0 mm, Härjedalen, Lövhögen, 1946-07-02, leg. N. Höglund, NHRS-COLE 00007432; 1 ♀ BL 11.0 mm, Torne lappmark, Silkimuotka, 1948-06-28, leg. N. Höglund, NHRS-COLE 00007433; 1 ♀ BL 11.0 mm, Torne lappmark, Silkimuotka, 1948-06-28, leg. N. Höglund, NHRS-COLE 00007438; 1 ♂ BL 10.0 mm, Torne lappmark, Silkimuotka, 1948-06-28, leg. N. Höglund, NHRS-COLE 00007436; 1 ♂ BL 11.0 mm, Lp. in., ex coll. Boheman, NHRS; 1 ♀ BL 11.2 mm, Lp. in., ex coll. Schönherr, NHRS; 1 ♀ BL 12.0 mm, Jämtland, ex coll. Rudolphi, NHRS; 1 ♂ BL 10.2 mm, Lp. i. S.U., NHRS. **Norway**: 1 ♂ BL 11.4 mm, 1 ♂ BL 10.9 mm, 1 ♂ BL 9.9 mm, 1 ♂ BL 10.1 mm, 1 ♀ BL 12.7 mm, 1 ♀ BL 13.5 mm HEN, Trysil: Ljørdalen, Skjærkjølen (EIS 65) 61°21'44.5"N, 12°40'06.3"E, 2014-VI-31, reared from *Salix lapponum*, Leg. T. Kvamme CTK; 1 ♂ BL 10.0 mm, BV, Ål: Vatsfjorden, 2006-07-17, leg. O. J. Lønnve, NHMO; 1 ♀ BL 12.5 mm, HEN, Trysil: Tangåtjønna, 2011-06-25, leg. P.K. Solevåg, CPKS; 1 ♂ BL 10.5 mm, OS, Nordre Land: Synfjellet, 1897-07-20/21, NIBIO; 1 ♀ BL 11.5 mm, HEN, Trysil: Ljørdalen, 2014-06-25, *Salix lapponum*, leg. Å. Lindelöw, CÅL; 1 ♀ BL 11.0 mm, 1 ♂ BL 11.0 mm and 1 ♂ BL 11.5 mm HEN, Skåret, RT90 6826517/1324435, 2014-06-25, *Salix lapponum*, leg. Å. Lindelöw, CÅL; 2 ♀♀ BL 12.5 mm, 1 ♀ BL 12.0 mm, 2 ♀♀ BL 13.0 mm, 1 ♀ BL 11.0 mm, 6 ♂♂ BL 11.0 mm, 1 ♂ BL 10.5 mm, 2 ♂♂ BL 10.0 mm, HEN, 5km NE Østby (Ljørdalen), 2014-05-31, reared from *Salix lapponum* (emerged 2014-06-12), leg. H. Wallin, CHW; 2 ♀♀ BL 13.0 mm and 1 ♂ BL 11.0 mm, HEN, 5km SE Trysil, 2014-05-31, reared from *Salix lapponum* (emerged 2014-06-08), leg. H. Wallin, CHW. **Finland**: 2 ♂♂ BL 10.0 mm, Enontekiö, 1951-08-26, leg. Hellman, MZH; 1 ♀ BL 10.3 mm, Enontekiö, 1951-08-26, leg. Hellman, NHRS; 1 ♂ BL 10.5 mm, Kemijärvi, 1936-06-22, leg. Krogerus, MZH; 1 ♀ BL 12.4 mm, Finland, ex coll. Schönherr, NHRS no. 8146 E94. **Russia**: 1 ♂ BL 10.0 mm, BW BL 2.5 mm, Central Russia (Russia Merid.), leg. Zarisin, ex coll. C. Nyberg, MZH: 1 ♂ BL 8.7 mm, Central Russia (Russia Merid.), ex coll. Duske, MZH; 1 ♂ BL 10.6 mm, Petsamo (Petjenga), leg. Hellén (id 716), MZH.

**Figure 8.**
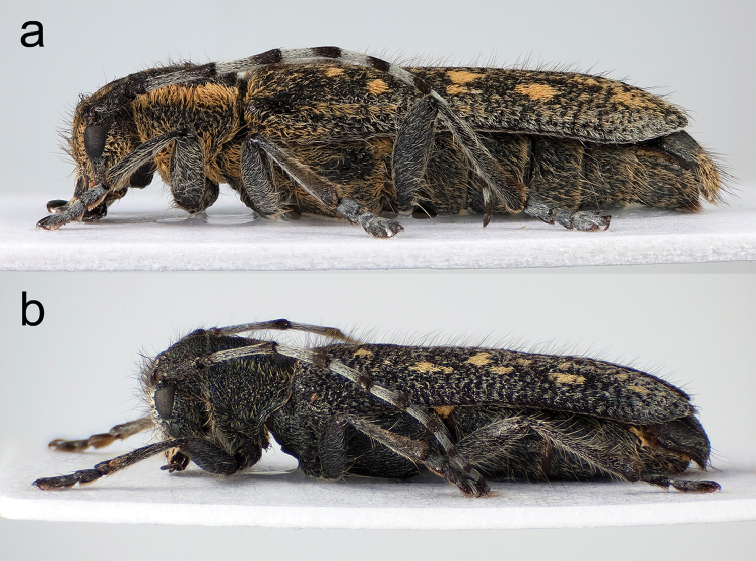
Habitus (lateral view). **a** *Saperda populnea populnea* (Linnaus, 1758), Stockholm, Nacka (Södermanland), Sweden (photo: Karsten Sund) **b** *S. populnea lapponica* ssp. n., Kiruna (Lappland), Sweden (photo: Karsten Sund).

##### Additional material examined.

The following specimens collected in Finland and available (through Boldsystems Public Data Portal) for photo examination includes: 1 ♀ COLFA181-10, Lapland, Inari, 1980-07-11, leg. Erkki Laasonen, id MP00443, ZMUO; 1 ♂ COLFA187-10, Lapland, Inari, 1993-08-26, leg. Juhani Itaemies, id MP00449, ZMUO.

##### Description.

A relatively small to medium-sized and subcylindrical subspecies with body length 9.5–13.0 mm in females and 8.0–12.0 mm in males, according to measurements from the present study. Body 3.1 times longer than wide in females and 3.4 times longer than wide in males (Fig. 6b–c, e–f). Integument black, the compressed pubescence is yellowish to whitish (most northern populations) (Figs 6c, f) to reduced orange-brown pubescence (southern populations) (Fig. 6b, e). Elytra with numerous long erected dark brown hairs. The pubescence in the southern populations is relatively dense in both sexes. The yellowish to whitish pubescence in the northernmost populations (above the Arctic Circle) is strongly reduced resulting in exposed and shining integument in both sexes. The orange-brown pubescence is present but weakly extended laterally in females from southern populations and the yellowish to whitish pubescence in females from northern populations very weak laterally (Fig. 8b).

**Figure 9.**
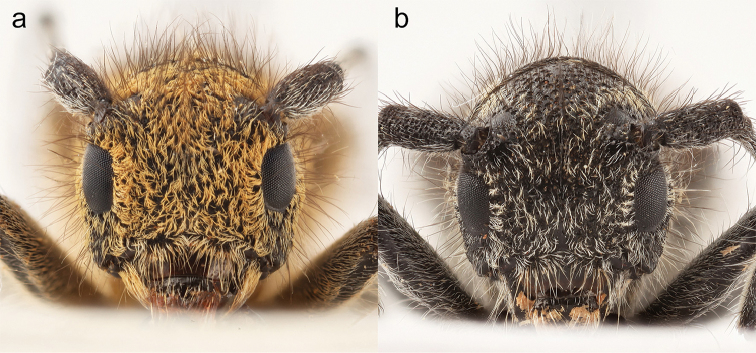
Frons. **a** ♀ *Saperda populnea populnea* (Linnaeus, 1758), Knutby (Uppland), Sweden **b** ♀ *S. populnea lapponica* ssp. n., Kiruna (Lappland), Sweden.

**Figure 10.**
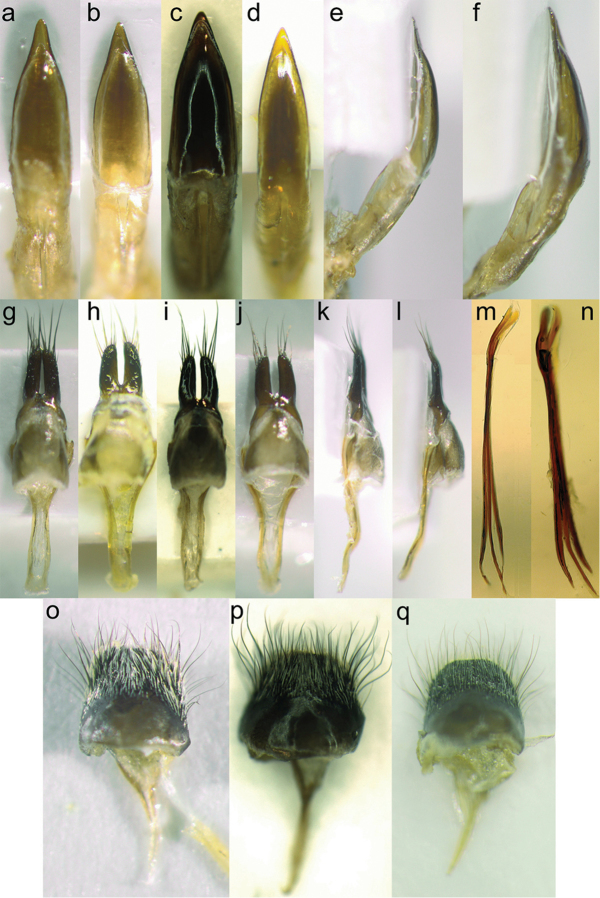
Aedeagi (**a–d** dorsal view **e–f** lateral view), parameres with median lobes (**g–j** dorsal view **k–l** lateral view), sclerite inside internal sac (**m–n**) and tergite VIII in males (**o–q**). **a** *Saperda populnea populnea* (Linnaeus, 1758), Joensuu, Finland **b** *S. populnea populnea*, Umeå (Västerbotten), Sweden **c** *S. populnea lapponica* ssp. n., Ljørdalen, Norway **d** Soppero (Lappland), Sweden **e** *S. populnea populnea* Joensuu, Finland **f** *S. populnea lapponica* ssp. n., Silkimuotka, Finland **g** *Saperda populnea populnea* (Linnaeus, 1758), Släp (Halland), Sweden **h** *S. populnea populnea*, Sillre (Medelpad), Sweden **i** *S. populnea lapponica* ssp. n., Ljørdalen, Norway **j** *S. populnea lapponica* ssp. n., Kittelfjäll (Västerbotten), Sweden; k: *S. populnea populnea*, Uppsala (Uppland) **l** *S. populnea lapponica* ssp. n., Enontekiö, Finland **m** *Saperda populnea populnea* (Linnaeus, 1758), Uppsala, Sweden **n** *S. populnea lapponica* ssp. n., Kiruna, Sweden **o** *Saperda populnea populnea* (Linnaeus, 1758), Uppsala, Sweden **p** *S. populnea lapponica* ssp. n., Trysil: Ljørdalen, Norway **q** *S. populnea lapponica* ssp. n., Kiruna, Sweden.

**Figure 11.**
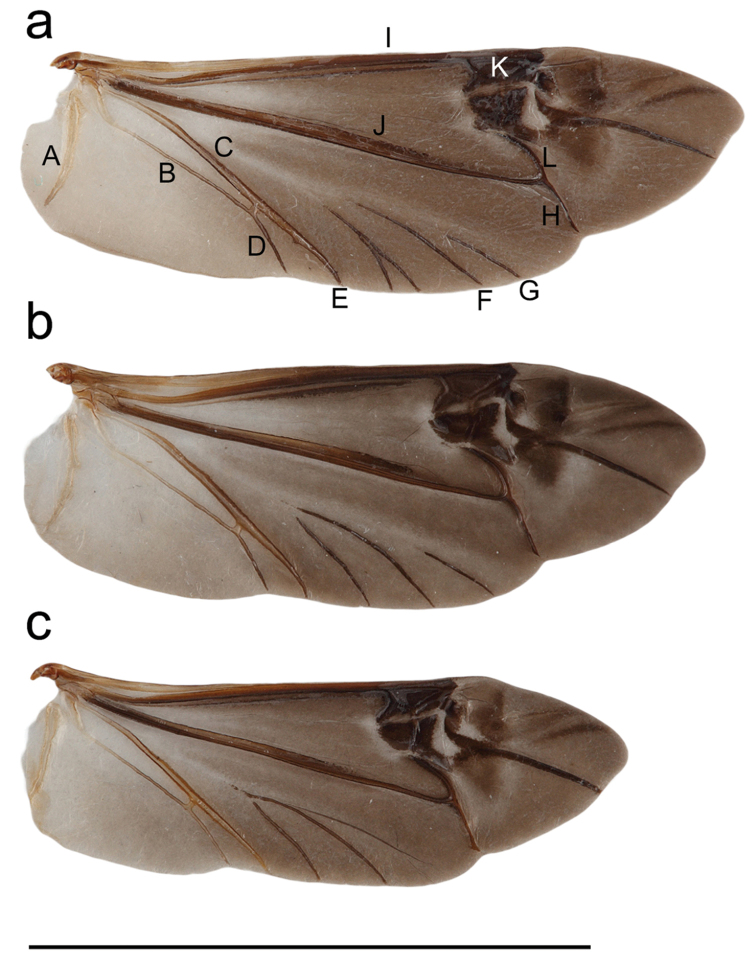
Hind wings. **a** ♀ *Saperda populnea populnea* (Linnaeus, 1758) reared from *Populus tremula* L., Uppland, Knivsta, Sweden. **A** AP vein **B** AA vein **C** CuA vein **D** AA3+4 vein **E** CuA3+4 vein **F** Mp4 vein **G** Mp3 vein **H** medial spur vein **I** RA vein **J** MP vein **K** radial cell **L** RP-MP vein **b** ♀ *Saperda populnea lapponica* ssp. n. reared from *Salix lapponum* L., Trysil: Ljørdalen Norway **c** ♀ *Saperda populnea lapponica* ssp. n. reared from *Salix lapponum* L., Luleå Lappmark, Gallugas 20 km W. Kiruna, Sweden. Scale bar 10 mm.

**Head in females.** Frons convex and broader than long (about 5 times broader than the width of one eye lobe), eyes with lower eye lobes slightly longer than broad and as long as gena below it. Genae posteriorly with long fringes of yellowish or whitish hairs and genae evenly narrowing towards mouthparts resulting in head being more “rounded” (Fig. 9b). Frons weakly covered with yellowish to whitish pubescence, and numerous dark brown, long and erected hairs. The area between antennal segments is shallowly impressed. Frons densely covered with orange-brown pubescence and numerous dark brown, long erect hairs. Genae posteriorly with long fringes of orange-brown hairs. **Head in males**: Frons convex and broader than long (about 4 times broader than the width of one eye lobe), eyes with lower eye lobes longer than broad and about 3 times longer than the short gena below. Head with frons rounded, genae straight and acutely narrowing towards mouthparts, frons weakly covered with whitish or orange-brown pubescence and numerous dark brown, long and erected hairs. Genae posteriorly with long fringes of orange-brown hairs. The area between antennal segments is shallowly impressed. **Mouthparts.** Frontoclypeal margin has a fringe of relatively long whitish pubescence and long, brown, suberect hairs. Clypeus glabrous except at base. Labrum with appressed, whitish pubescence and numerous long, suberect, orange-brown setae. **Antennae.** Short, slender, at the most extending beyond the middle of elytra by 2–3 antennomeres in females (Fig. 6b–c). In males, the antennae reach by 3-4 antennomeres past the middle; thus, antennae are always shorter than body in males (Fig. 6e–f). The segments from third segment are annulate. Annulation on antennal segments greyish and covering about ¾ of the anterior part of each antennal segment. The subconical, third segment is longer than first and fourth. Scape slender and coarsely punctured with a combination of large and small, shallow punctures and long black hairs. **Thorax.** Pronotum subcylindrical, slightly broader than long, lacking lateral spines. Pronotal disk convex, weak median line often with a glabrous and shining area medially, base shallowly impressed, coarse punctures except medially, densely covered with long erect and brown hairs, two broad lateral yellowish stripes with a weak median line interrupted medially. Prosternum densely pubescent with yellowish and whitish hairs. **Elytra.** 2.6–3.0 times longer than broad in females and 2.7–3.1 times longer than broad in males. No distinct carinae present on elytra. Parallel and weakly narrowing towards apices, apices narrowing and rounded, punctures coarse, deep, contiguous towards humeri and apices and confluent medially (especially in males where confluent punctures form short and weakly raised ridges transversally on each elytron), pubescence relatively weak to dense. There are generally eight relatively distinct and small to relatively large, yellowish to whitish spots on elytra, arranged in pairs: the first and third near the suture, spots in the third pair often elongated transversally or even divided into two spots each, spots in the fourth pair elongated transversally and placed on the middle of elytra in females (Fig. 6b–c), Females from northern populations have irregular spots of yellowish to whitish pubescence between the third and fourth pair of spots and towards apices. No missing spots were seen in any of the examined specimens, but a few old worn specimens had very small i.e. obsolete spots on the elytra. The remaining part of elytra is covered with scattered yellowish or whitish pubescence and numerous long brown hairs. **Scutellum.** “U-shaped” and weakly covered with whitish hairs (southern populations) or entire scutellum glabrous (most northern populations). **Hind wing.** About 11.0 mm long in females and 9.0 mm long in males (Fig. 11b–c). Covered with weak smoky tint. Several veins are broken with apical portions not connected to basal portions. MP3 (rudimentary), MP4 and AA vein broken. Radial cell very strong and complete. **Legs.** Relatively short, densely covered with fine whitish pubescent including tarsi, tarsal claws lacking a process. **Venter.** Densely covered with whitish to yellowish pubescence in both sexes, prosternal process narrow and flattened anteriorly. Mesosternum and abdominal ventrites are densely covered with yellowish or whitish pubescence and numerous yellowish and long, erected hairs. Posterior margin of sternite VII rounded and often deeply notched on medially. **Male terminalia.** Aedeagus 2.0-2.3 mm long, evenly curved towards apex and compressed dorso-ventrally (Fig. 10f), dorsal surface smooth and shining with apical part weakly narrowed towards apex (Fig. 10c–d). Tegmen with parameres 2.1–2.5 mm longer and straight dorso-ventrally (Fig. 10l). Parameres acutely narrowing towards apex, with dorsal surface glabrous and shining, or (rarely) with entire surface densely covered with punctures and suberected setae. The inner margins well-separated and diverging towards apices (Figs 10i–j). Tergite VIII 0.6–1.0 mm long, relatively large and rounded with the posterior margin concave in the middle and densely covered with white pubescence and numerous long brown hairs (Fig. 10p-q). Sclerites inside internal sac 1.7–2.1 mm long consisting of three parallel “shaft-like” structures, of which the apical end (top) is elongated and posterior end blunt and acutely narrowing towards posterior end (Fig. 10n). The colour of male genitalia is yellowish to dark brown. **Female terminalia.** Tignum almost straight, 6.5–8.2 mm long (width 0.1–0.2 mm at the widest point apically). Tergite VIII posterior margin (width: 1.0 mm) with a few brown hairs. The colour is brown. Spermathecal capsule: strongly sclerotised, yellowish, round and supplied with a short shaft, diameter: 0.5 mm.

##### Remarks.

morphological characteristics are mainly based on type specimens, either collected on, or reared from branches of *Salix lapponum*. *S. populnea lapponica* ssp. n. is separated from *S. populnea populnea* by the overall smaller body size, shorter antennae in both sexes, reduced pubescence on thorax and elytra, mainly yellowish to whitish pubescence, reduced or absent pubescence on scutellum and short frons in females which is giving the appearance of a rounded head (Fig. 8b). The characters presented herein are mainly based on newly hatched and fully sclerotised specimens. Small, dark and less pubescent specimens are easily recognized in collections in Fennoscandia and were in most cases, found to belong to the new subspecies *S. populnea lapponica* ssp. n. There are variations in the body size and colour pattern on elytra between the various populations of *S. populnea lapponica* ssp. n. The slightly larger specimens occurring in the southern populations near Trysil, Norway, have more distinct spots on elytra. The darker and smaller specimens from the northern populations, occurring in the northern Scandinavian mountain range near e. g Kiruna, also have intermediate forms occurring e.g. in Juksjaur near Tärnaby. The darker and slightly smaller specimens have more reduced spots on elytra. No such geographical variation in body size and colour pattern has been found in *S. populnea populnea* in Fennoscandia.

##### Etymology.

The name is an adjective used as a substantive in the genitive case derived from the specific name of the host plant *Salix lapponum*.

##### Distribution.

The distribution of *S. populnea lapponica* ssp. n. is within the distribution of *Salix lapponum* in Fennoscandia (Hultén 1971). The most southern populations of *S. populnea lapponica* ssp. n. occur near Trysil, Norway, while the most northern populations occur north of the Arctic Circle (Fig. 13). Since *Salix lapponum* is distributed eastwards in Siberia approximately to the Jenisej Valley (Hultén and Fries 1986), it is possible that *S. populnea lapponica* ssp. n. has a much wider distribution in Russia than we are able to show in the present paper.

##### Biology.

**Figure 12.**
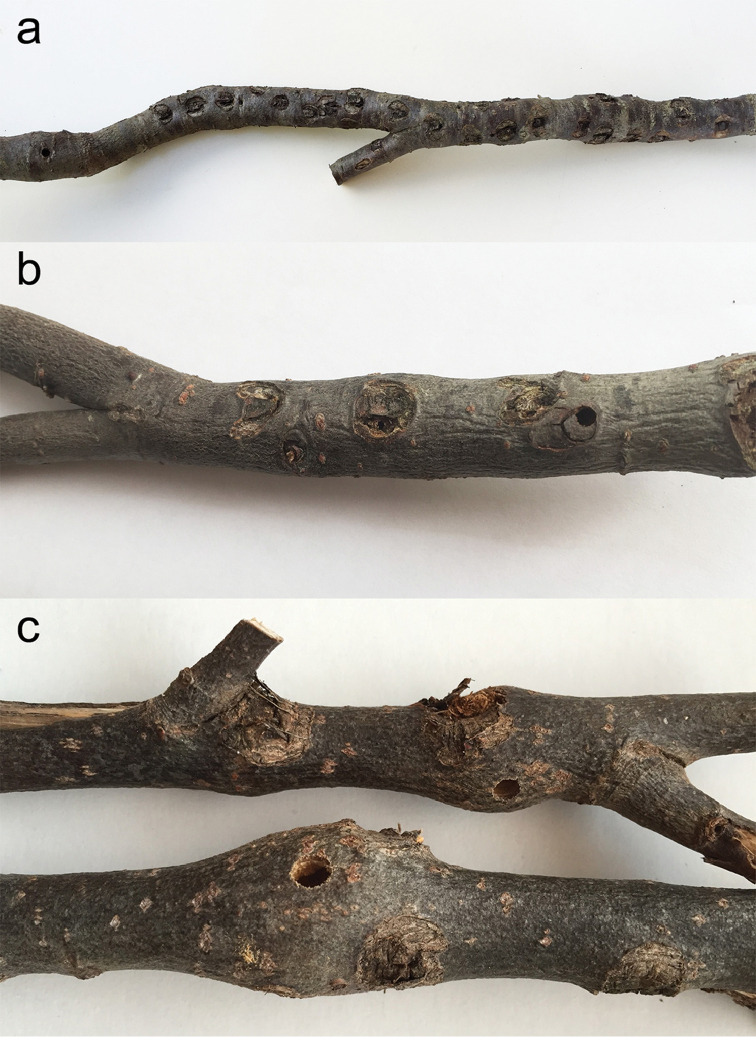
Host tree attacks. **a** extensive attacks of *Saperda populnea lapponica* ssp. n., on the entire stem and branches of *Salix lapponum* L. from Trysil: Ljørdalen, Norway **b** three adjacent attacks, including an exit hole, of *Saperda populnea lapponica* ssp. n., on a stem of *Salix lapponum* L. from Gällivare (Lappland), Sweden **c** single attacks, including exit holes, of *Saperda populnea populnea* (Linnaeus, 1758), on a stems of *Populus tremula* L. (beetles emerged at top: male, bottom: female), from Knivsta (Uppland), Sweden.

The attacks are similar to *S. populnea populnea* where females form a “U-shaped lid” in the bark under which an egg is deposited. Stems and branches around 1–2 cm in diameter are used. However, normally no galls are formed by the host tree (Fig. 12a–b). The attacks can be massive and one single stem can contain up to 30 attacks (Fig. 12a). Larvae can live during a number of consecutive years since old exit holes are present together with live larvae. It is, therefore, likely that several generations of beetles can develop within the same stem of *Salix lapponum*. Exit holes are normally slightly larger when made by female beetles compared to male, reflecting the differences in size and shape. The development takes at least 2 years, since both small and full-grown larvae were found in stems of *Salix lapponum* after adults had emerged. The localities are wetter than localities where *S. populnea populnea* are found, since *Populus tremula* do not occur in biotopes where *S. lapponum* occur. As a consequence, *S. populnea populnea* and *S. populnea lapponica* ssp. n. live in well separated habitats.

In addition, parasites including wasps and flies frequently attack *S. populnea populnea* (Schwenke 1974, Pulkinn and Yang 1984, Georgiev 2001). Very few such parasites have been collected from stems attacked by *S. populnea lapponica* ssp. n. which might be due to climatic factors. However, we did recover two parasitoid wasps of the family Ichneumonidae from downy willow hatching wood with *Saperda populnea lapponica* ssp. n. attacks. These were identified as one *Poemenia hectica* (Gravenhorst, 1829) (Poemeniinae) and one Campopleginae, possibly belonging to the genus *Pyracmon* (det. Jacek Hilszczański). Unfortunately, the second specimen was damaged during post transfer and could therefore not be identified with certainty. While Campopleginae includes species known as parasitoids of saproxylic beetles, *Poemenia* is known as a parasitoid of wood-nesting wasp larvae, so that it may not have been (directly) related to the *Saperda populnea lapponica* ssp. n. larvae.

**Figure 13.**
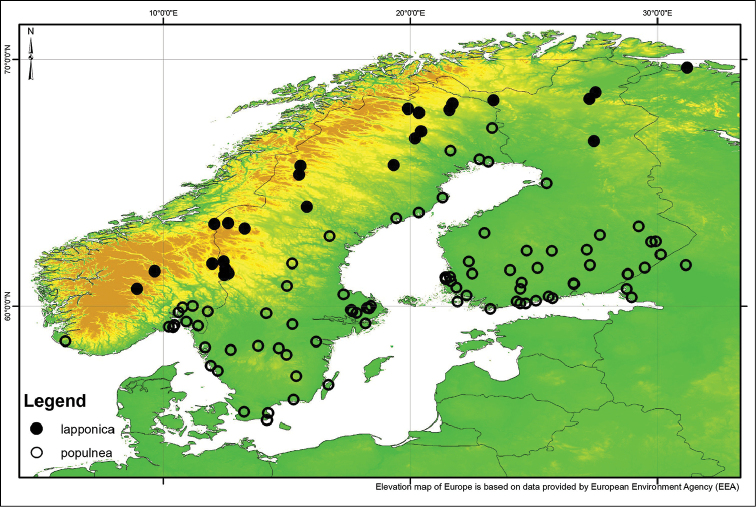
Distribution of records mainly from Fennoscandia. Open circles: *Saperda populnea populnea* (Linnaeus, 1758) and black dots: *S. populnea lapponica* ssp. n.

**Figure 14.**
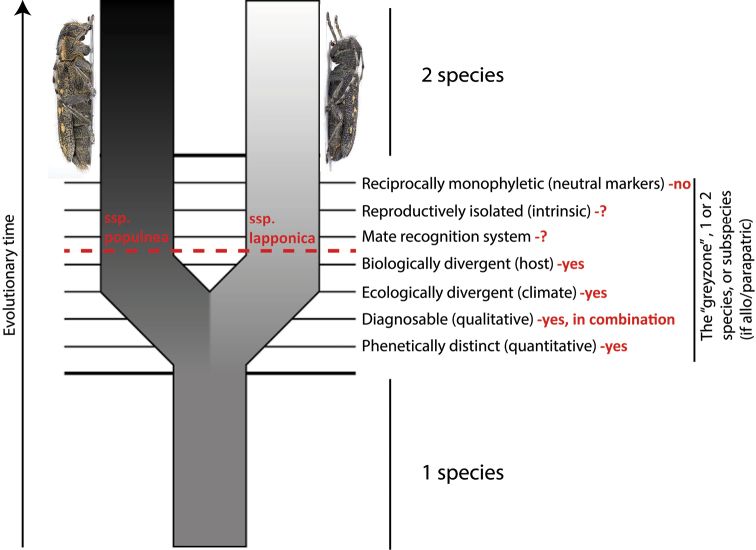
Subspecies of *Saperda populnea* (Linnaeus, 1758) fall in the grey zone under the unified species concept. Adapted from de Quieroz (2007) and beetle photos by Karsten Sund.

## Discussion

The new subspecies *S. populnea lapponica* ssp. n. is relatively similar to *S. populnea balsamifera* stat. n. According to the original description, *S. populnea balsamifera* stat. n. is characterised by narrow body and weak pubescence with very small dots on elytra. There is no information on body length in the original description by Motschulsky (1860). Cherepanov (1991) redescribed *Compsidia balsamifera* by referring to the very pubescent and orange-brown form, 11–13 mm long, found on *Salix* near Novosibirsk. According to M.L. Danilevsky (pers. comm.), the pubescent and orange-brown form is very rare, whereas the darker and less pubescent form with small dots on elytra is more common. The examined specimens of *S. populnea balsamifera* stat. n. differ from *S. populnea lapponica* ssp. n. in the following characters: antennae relatively long in both sexes in *S. populnea balsamifera* stat. n. (extending beyond the middle of elytra by 4.5 antennomeres in males), antennae relatively short in both sexes in *S. populnea lapponica* ssp. n. (extending beyond the middle of elytra by 3.5 antennomeres in males); head in female in *S. populnea balsamifera* stat. n. long (almost “square-formed”) and lower eye lobe as long as gena below it, head in female in *S. populnea lapponica* ssp. n. rounded, and lower eye lobe 2–3 times longer than the short gena below it; scutellum in *S. populnea balsamifera* stat. n. covered with pubescence, scutellum in *S. populnea lapponica* ssp. n. glabrous or at most weakly covered with few hairs; the four pair of dots on elytra in *S. populnea balsamifera* stat. n. very small and rounded, the four pair of dots on elytra in *S. populnea lapponica* ssp. n. larger and often elongated transversally (third pair of dots); male aedeagus in *S. populnea balsamifera* stat. n. very short and almost straight, aedeagus in *S. populnea lapponica* ssp. n. long and evenly curved; parameres in *S. populnea balsamifera* stat. n. short and weakly narrowing towards apices, parameres in *S. populnea lapponica* ssp. n. long and acutely narrowing towards apices; tergite VIII in *S. populnea balsamifera* stat. n. short and weakly supplied with very fine hairs, tergite VIII in *S. populnea lapponica* ssp. n. long and densely covered with pubescence; sclerite inside internal sac in *S. populnea balsamifera* stat. n. very short with posterior end separated (“V-formed”), sclerite inside internal sac in *S. populnea lapponica* ssp. n. long with posterior end blunt. The examined specimens of *S. populnea balsamifera* stat. n. also differ from *S. populnea populnea* in several of the above-mentioned characters. Thus, we agree with Danilevsky (2016, word document on website) that it cannot be regarded as a synonym of *Saperda populnea* and we here formally elevate *S. balsamifera* to a separate subspecies: *S. populnea balsamifera* stat. n. It may very well be that it should be recognised as a full species, but more material is needed to examine the variation in characters. The type of *S. populnea balsamifera* stat. n. (which appears to be a male) represents a “black” form with almost glabrous elytra, apart from the very small but distinct spots on elytra not seen in *S. populnea lapponica* ssp. n. Whether the pubescent orange brown form and the darker less pubescent form truly are conspecific also needs further investigations. *Saperda populnea balsamifera* stat. n. is only known from Siberia and Far East of Russia, China and Japan (Löbl and Smetana 2010). The type locality is in Mongolia, collected on *Populus balsamifera* L. It appears that all records of *S. populnea balsamifera* stat. n. are outside the range of *Salix lapponum* according to the map presented by Hultén E and Fries M (1986). Our findings indicate that the western subspecies *S. populnea lapponica* ssp. n. is more closely related to *S. populnea populnea* than to the eastern subspecies *S. populnea balsamifera* stat. n. We also follow Löbl and Smetana (2010) and tentatively consider *S. innotatipennis* Pic, 1910 (Fig. 7a) to be synonymous with *S. populnea balsamifera* stat. n., although further studies are required to fully investigate the relationship between *S. populnea balsamifera* stat. n. and *S. innotatipennis*.

We agree with Shapovalov (2013) and (Bezark 2016) that the North American species *S. moesta moesta* Le Conte is a valid species, and that *S. moesta tulari* (Le Conte) is a valid subspecies (Bezark 2016). *S. moesta moesta* and *S. moesta tulari* are easily distinguished from *S. populnea populnea* and *S. populnea lapponica* ssp. n. by the deep contiguous or scattered punctuation and lack of spots on elytra in both sexes. Further studies are required to fully investigate the relationship between *S. moesta moesta* and *S. moesta tulari*. *Saperda populnea populnea* was earlier supposed to occur in North America (Felt and Joutel 1904), but it has been corrected in recent work (Linsley and Chemsak 1995, Bezark 2016).

*Saperda gilanense* was described based on specimens from Northern Iran (Shapovalov 2013). The species differ from *S. populnea populnea* by the very bright yellowish and rounded spots on elytra. We have only examined two paratypes (male/female), and further studies are required, preferably including DNA data, to fully evaluate the taxonomic status of the species.

The remaining species within the subgenus subgenus Compsidia include *S. bacillicornis* Pesarini & Sabbadini, 1996, *Saperda bilineatocollis* Pic, 1924 and *S. messageei* Breuning, 1962.

*S. bacillicornis* is easily separated from *S. populnea populnea* by the narrow and dorso-ventrally flattened prothorax and the antennal segments uniformly covered with a whitish pubescence from 3^rd^ antennomere and not annulated. *S. bilineatocollis* (Fig. 7b) is distinguished from *S. populnea populnea* by the absence of spots on elytra and the distinct and broad longitudinal orange-brown stripe on elytra. The lower eye lobe on the HT female of *S. bilineatocollis* is as long as the gena below it. *S. bilineatocollis* occur in Far East of Russia and in China (Löbl and Smetana 2010). DNA of *S. bilineatocollis*, based on the genbank sequence for which we have seen a photo of the voucher specimen, was only slightly different (about 2.09–2.60%) from *S. populnea* (Fig. 3). Thus, further studies are required to fully investigate the relationship between *S. bilineatocollis* and *S. populnea populnea* and whether they do occur sympatrically in Far East Russia and China. Here, we do consider *S. bilineatocollis* to be a valid species. *S. messageei* is very similar to *S. populnea populnea* and the question is if this is a mislabelled specimen or even an introduced specimen to Laos. Similarly, we found an old specimen of *S. populnea populnea* labelled “Java”. None of these four species (*S. gilanense*, *S. bacillicornis*, *S. bilineatocollis*, *S. messageei*) are, however, similar to *S. populnea lapponica* ssp. n.

The male genitalia of all other Palaearctic species of *Saperda* differ from both *S. populnea populnea* and *S. populnea lapponica* ssp. n. Each species has unique male genitalia, although the male genitalia appear to be relatively similar between *S. carcharias* and *S. similis*. These two species also had a relatively small genetic distance (2.59%). The most different and striking sclerites inside the internal sac are found in *S. scalaris*, where they exhibit a broad and “fork-shaped” structure. We found no difference in hind wing morphology between *S. populnea lapponica* ssp. n. and *S. populnea populnea*, although statistical analysis with the use of selected landmarks on hind wings has been applied to differentiate two other cerambycid species: *Leiopus nebulosus* L. and *L. linnei* Wallin et al., 2009 (Rossa et al. 2017).

The other species synonymised by Löbl and Smetana (2010) and aberrations earlier synonymised by Breuning (1966) are all considered to be variations of *S. populnea populnea* with reduced number of spots on elytra of which several have been included as drawings by Villiers (1978). The synonymised species include *Leptura betulina* Geoffroy, 1785, S. ab. bickhardti Sattler, 1918, *Cerambyx decempunctatus* DeGeer, 1775, S. f. kavani Roubal, 1933, *S. populi* Duméril, 1860, S. ab. quadripunctata Podaný, 1953 and *S. salicis* Zetterstedt, 1818. No such reduction in the number of spots on elytra has been found in *S. populnea lapponica* ssp. n.

*S. salicis* was described from specimens collected on *Salix viminalis* L. at Abusa near Lund in southern Sweden (Zetterstedt 1818). A lectotype of *S. salicis* has been designated and it corresponds to the original description. Later, Zetterstedt (1828, 1840) referred to small and dark specimens rarely collected by himself in the Swedish Lappland earliest in 1820 (Lycksele and Umeå Lappmark), but without any species or subspecies description. Gyllenhal (1827) mentioned *S. salicis* as a southern species and called it “var. b”. The two specimens of *S. populnea lapponica* ssp. n. labelled “Zetterstedt” and preserved in the Leonard Gyllenhal collection at UUZM must have been collected by Johan Wilhelm Zetterstedt in the Swedish Lappland. It is known, from preserved letters between these two entomologists, that Zetterstedt visited Gyllenhal when he returned from his journeys to Lappland. We, therefore, assume that the two northern specimens were given to Gyllenhal on one of these occasions. Roubal (1933) and Ehnström and Holmer (2007) incorrectly assumed that *S. salicis* was the boreal form of *S. populnea populnea*. In more recent years Ehnström and Axelsson (2002) wrote (page 312): “*The specimens living in the mountains are so clearly different from other specimens that they might be a separate species*” [translated from Swedish] and Heliövaara et al. (2004) mentioned: “*A darker and more slender morph (possibly a separate species), which lives on Salix lapponum, is more abundant in the northern parts of the country*”. However, no species or subspecies description was made.

That *Saperda populnea populnea* and *Saperda populnea lapponica* ssp. n. were not reciprocally monophyletic (Figs 2–3) by a neutral marker like COI was not surprising (see Patten 2010, Zink 2004). It is clear that reciprocal monophyly should not be the null expectation for subspecies (Patten 2010, 2015). Reciprocal monophyly in neutral markers is mainly related to the time since divergence and may take a very long time, dependent on effective population size (Zink 2004; Bergsten et al. 2012). It is also possible that these two subspecies hybridize and still maintain some gene flow at the contact zone. Maintained reproductive compatibility is part of the classical definition of a subspecies outlined by Ernst Mayr with small variations in several of his landmark books: Mayr (1942: 106): “*The subspecies, or geographical race, is a geographically localized subdivision of the species, which differs genetically and taxonomically from other subdivisions of the species*”; Mayr (1963: 348): “*A subspecies is an aggregate of local populations of species, inhabiting a geographic subdivision of the range of the species, and differing taxonomically from other populations of the species*”; Mayr (1969: 41): “*A subspecies is an aggregate of phenotypically similar populations of a species, inhabiting a geographic subdivision of the range of the species, and differing taxonomically from other populations of the species*”. Further, as the subspecies definition was subordinate the species under the Biological Species Concept (BSC) paradigm, then “*Because they are below the species level different subspecies are reproductively compatible*” (O’Brien and Mayr, 1991: 1188).

The trinomial subspecies remain a contentious hierarchical level in zoological taxonomy (Zink 2004). Some authors argue for the abandonment of the concept altogether (Wilson and Brown 1953), but it is formally recognized by the International Commission on Zoological Nomenclature (ICZN 1999), albeit without giving any advice or criteria for its recognition. The concept is variously used in different disciplines, extensively in mammals and birds (Gippoliti and Amori 2007, Mayr 1982), less so in insects (Haigh et al. 2006) in general, but more commonly in some groups like butterflies (Braby et al. 2012, Gillham 1956). The concept is more than a mere academic debate as subspecies are recognized in various red-lists and conservation programs, and hence the recognition as a subspecies or not can have legal and monetary consequences (Haigh et al. 2006, Braby et al. 2012, O’Brien and Mayr 1991, Gippoliti and Amori 2007). There have been a few attempts to put a quantifiable limit on what a subspecies is. The most well known such threshold is the “75% rule” (Amadon 1949, Patten 2010, Patten and Unitt 2002): members of a subspecies should be diagnosable by some character so that at least 75% of individuals in subspecies A should be outside of the distribution of 99% of individuals of subspecies B. Patten and Unitt (2002) formalised this rule in a simple t-test statistics. Another suggestion of a subspecies definition in the age of genetic data was proposed by Patten (2015): “*I propose that under the phylogenetic species concept, a (morphologically) diagnosably distinct, geographically circumscribed clade that does not form a distinct (neutral) genetic cluster or is not reciprocally monophyletic (I mention this because its assessment is common practice, not because it is a criterion inherent to the concept) in relation to other such clades be deemed a subspecies and not a species*”.

*S. populnea populnea* and *S. populnea lapponica* ssp. n. fit this definition perfectly. However, we believe that while authors are proposing various new subspecies definitions (Braby et al. 2012, Patten 2015, Patten and Unitt 2002, O’Brien and Mayr 1991), the same mistake of confusing what subspecies are and how they can be recognized (operational criteria) is repeated, as with the century old species concept debate. That debate was solved by separating the necessary properties (the definition) from the secondary operational criteria in the Unified Species Concept (USC) (de Quieroz 2007). Species under the USC are separately evolving metapopulation lineages (de Quieroz 2007). That is the only necessary property of species. Subspecies under the USC are basically recognized in the grey-zone, commonly displaying some, but not all, properties that may define separately evolving lineages (Braby et al. 2012) (Fig. 14). There is unanimous agreement in all subspecies definitions that subspecies are 1) geographically defined and 2) diagnosable by at least one presumably heritable character. The meaning of geographically defined may vary, and some restrict the use to allopatric, but not parapatric situations (Braby et al. 2012). Also the meaning of diagnosable may vary, whether focused on difference in mean or degree of overlap (Patten and Unitt 2002), and whether one or multiple concordant characters should be required (O’Brien and Mayr 1991). There is also unanimous agreement that subspecies are potentially, but not necessarily, incipient species (Mallet 2001, Patten 2010, O’Brien and Wilson 1991, Crusz 1986). Species evolving through allopatric speciation basically go through a stage which we would call a subspecies (Mayr 1942). From that does not follow, however, that all subspecies become full species with time (Patten 2010, O’Brien and Mayr 1991). A subspecies may also merge back with say, its sister subspecies through geneflow at secondary contact (Patten 2010), or go extinct. We therefore propose that under the unified species concept, subspecies are defined as potentially incipient species in allopatry or parapatry that are diagnosable by at least one presumably heritable trait. Hence the only necessary properties of subspecies are that they are potentially incipient species under the USC (.i.e. potentially on their way to become separately evolving metapopulation lineages), they are currently diagnosable by at least one trait that is heritable and not environmentally determined, and that they are geographically defined. Reciprocal monophyly or not in neutral markers, quantitative thresholds like the 75% rule, reproductive compatibility or degree of gene flow should not be part of the definition.

## Supplementary Material

XML Treatment for Saperda populnea populnea

XML Treatment for Saperda populnea lapponica
